# RpoN/Sfa2-dependent activation of the *Pseudomonas aeruginosa* H2-T6SS and its cognate arsenal of antibacterial toxins

**DOI:** 10.1093/nar/gkab1254

**Published:** 2021-12-20

**Authors:** Luke P Allsopp, Alice C Z Collins, Eleanor Hawkins, Thomas E Wood, Alain Filloux

**Affiliations:** Department of Life Sciences, MRC Centre for Molecular Bacteriology and Infection, Imperial College London, London, UK; National Heart and Lung Institute, Imperial College London, London, UK; National Heart and Lung Institute, Imperial College London, London, UK; Department of Life Sciences, MRC Centre for Molecular Bacteriology and Infection, Imperial College London, London, UK; Department of Life Sciences, MRC Centre for Molecular Bacteriology and Infection, Imperial College London, London, UK; Department of Life Sciences, MRC Centre for Molecular Bacteriology and Infection, Imperial College London, London, UK

## Abstract

*Pseudomonas aeruginosa* uses three type six secretion systems (H1-, H2- and H3-T6SS) to manipulate its environment, subvert host cells and for microbial competition. These T6SS machines are loaded with a variety of effectors/toxins, many being associated with a specific VgrG. How *P. aeruginosa* transcriptionally coordinates the main T6SS clusters and the multiple *vgrG* islands spread through the genome is unknown. Here we show an unprecedented level of control with RsmA repressing most known T6SS-related genes. Moreover, each of the H2- and H3-T6SS clusters encodes a **s**igma **f**actor **a**ctivator (SFA) protein called, Sfa2 and Sfa3, respectively. SFA proteins are enhancer binding proteins necessary for the sigma factor RpoN. Using a combination of RNA-seq, ChIP-seq and molecular biology approaches, we demonstrate that RpoN coordinates the T6SSs of *P. aeruginosa* by activating the H2-T6SS but repressing the H1- and H3-T6SS. Furthermore, RpoN and Sfa2 control the expression of the H2-T6SS-linked VgrGs and their effector arsenal to enable very effective interbacterial killing. Sfa2 is specific as Sfa3 from the H3-T6SS cannot complement loss of Sfa2. Our study further delineates the regulatory mechanisms that modulate the deployment of an arsenal of T6SS effectors likely enabling *P. aeruginosa* to adapt to a range of environmental conditions.

## INTRODUCTION

Interbacterial competition enables strains to outcompete their rivals and dominate in a broad range of niches giving them the competitive edge for survival. *Pseudomonas aeruginosa* harbors three T6SS clusters and multiple orphan T6SS islands (*vgrG*, *paar*, *hcp*, toxin/immunity), which have been shown to mediate host manipulation and interbacterial competition (1,2). Multiple regulators converge to control these systems. However, RsmA has been shown to post-transcriptionally repress genes from all three clusters and multiple orphan islands (3).

To live long and prosper in diverse environments bacteria must sense and respond to environmental signals. Such signal integration leads to optimal use of resources, controls motility, virulence factor production and triggers defensive and offensive strategies (1,4,5). Transcriptional control is the most efficient way for bacteria to respond. One critical regulator that facilitates this in Gram-negative bacteria is RpoN.

RpoN is the major alternative sigma factor and is historically linked with enabling nitrogen metabolism but has increasingly been shown to regulate many surface-expressed proteins and virulence factors (6). RpoN is essential for full bacterial virulence in a range of plant and animal models and plays a critical role in controlling flagella-based motility (7,8). For activation, open complex formation and subsequent mRNA production, RpoN relies on enhancer binding proteins (EBP) also known as sigma factor activator (SFA) proteins. For example, in the case of flagella, the EBP FleQ is the major regulator which works in conjunction with RpoN to activate most flagella-associated genes (7).

In addition to controlling motility, RpoN has been associated with controlling T6SS clusters in multiple organisms including *Vibrio cholerae, P. aeruginosa*, and *Klebsiella pneumoniae* amongst others (9–14). RpoN has been reported to affect the *P. aeruginosa* H2- and H3-T6SS operons (10,12,13). Here, we postulated that RpoN-mediated regulation could enable coordinated control of all the T6SS clusters and orphan *vgrG* islands in *P. aeruginosa*. In contrast to a previous report (12) we show this is indeed the case through using a combination of RNA-seq, reporter fusions, ChIP-seq, western blot analysis, secretion assays and killing assays. We show that RpoN is required for robust expression of the genes in the core H2-T6SS operon. However, RpoN control revealed a greater regulatory variation of these systems as components of the H1-T6SS and H3-T6SS are repressed. Strikingly, RpoN-dependent regulation of H2-T6SS genes extends beyond the central cluster as it coordinates expression of the orphan H2-T6SS gene islands spread throughout the genome. Each of these H2-T6SS clusters that are controlled contain a *hcp2* homologue (PA14 has four *hcp2* genes, *hcp2ABC* that share 97.5% sequence identity) followed by a *vgrG* gene (*vgrG14*, *vgrG2a*, *vgrG2b*, *vgrG6*). We also demonstrate that RpoN works in conjunction with the sigma factor activator 2 (Sfa2) to activate the H2-T6SS cluster promoter and orphan gene clusters, enabling coordinated expression of an arsenal of T6SS armaments for optimal antibacterial activity. Furthermore, we show that deletion of *rpoN* results in overexpression of a H3-T6SS component, Hcp3, highlighting inverse regulatory control between the H2-T6SS and H3-T6SS. Finally, we show specificity in such control as *sfa3* encoded in the H3-T6SS cluster cannot rescue deletion of *sfa2*. Thus, our study defines how *P. aeruginosa* controls and coordinates deployment of its T6SS arsenal, which will be very valuable in designing strains with variable T6SS potencies and assess how these systems impact bacterial behavior in a polymicrobial context.

## MATERIALS AND MEHTODS

### Biological resources and growth conditions

Bacterial strains used in this study are listed in Supplementary Table S1 and plasmids in Supplementary Table S2. Strains were grown in or on tryptone soy broth (TSB), lysogeny broth (LB) or Vogel-Bonner medium (VBM) (20 mM magnesium sulfate heptahydrate, 200 mg anhydrous citric acid, 1 g potassium phosphate dibasic and 350 mg ammonium sodium phosphate dibasic tetrahydrate) or terrific broth (TB) with agitation at either 25°C or 37°C with the addition of agar as required. Antibiotics were supplemented to the media as appropriate. For *P. aeruginosa* the following antibiotic concentrations were used: streptomycin 2000 μg/ml, tetracycline 50–150 μg/ml, rifampicin 75 μg/ml, and gentamycin 40–100 μg/ml and for *Escherichia coli*: streptomycin 50 μg/ml, tetracycline 15 μg/ml and kanamycin 50 μg/ml).

### DNA manipulation

Genes or mutator fragments in this study were amplified via polymerase chain reaction (PCR) using KOD Hot Start DNA Polymerase (Novagen) in the presence of Betaine (Sigma) or standard Taq polymerase (NEB) with DMSO (Sigma) using *P. aeruginosa* PA14 or PAO1 as the template. Primers used are listed in Supplementary Table S3. DNA isolation was performed using the PureLink Genomic DNA mini kit (Life Technologies). Isolation of plasmid DNA was performed using the Monarch Plasmid Miniprep Kit (NEB) or QIAprep spin miniprep kit (Qiagen). Restriction endonucleases were used according to the manufacturer's specifications (NEB). DNA sequencing was performed by GATC Biotech. Complementation was performed using miniCTXplac (15).

### *Pseudomonas* mutant construction

*Pseudomonas aeruginosa* deletion mutants were constructed as previously described (16). Briefly, PCR followed by splice overlap extension PCR generated DNA fragments with in-frame deletions of the *P. aeruginosa* genome. These mutator fragments were cloned into the suicide vector pKNG101. After mobilisation into *P. aeruginosa* by three-partner conjugation from *E. coli* CC118λ*pir* with the 1047 pRK2013 helper strain, transconjugants were selected on VBM supplemented with 1.5% (w/v) agar and streptomycin. Next, counter-selection on 20% (w/v) sucrose LB agar plates at ambient temperature for 72 h led to plasmid loss and generated double recombinants. All mutants were confirmed with external primers (Supplementary Table S3). A similar strategy was used to engineer the wild-type *rpoN, sfa2 and sfa3* genes to encode for a triple Flag epitope tag at the C-terminal end of each protein (Supplementary Table S3).

### RNA-seq

All strains were grown overnight and sub-cultured in TSB. Strains were grown at 37°C to log phase (OD_600 nm_ of 0.8). Volumes containing 0.75 OD units (∼10^8^ bacteria) of each sample were collected, spun down, and resuspended in directly 100 μl of formamide (95%) containing 1% β-mercaptoethanol, 18 mM EDTA and 0.025% SDS. Three biological samples were performed for each strain. The samples were then incubated at 95°C for 7 min, cooled on ice and snap frozen. A serial dilution of each sample from 1 to 1 × 10^–8^ was performed in parallel and plated onto LB agar. Cell pellets were shipped on dry ice to Vertis Biotechnologie AG for extraction and downstream processing similar to that previously performed (17). Briefly, the RNA extracted was examined by capillary electrophoresis before rRNA depletion, adapter ligation to 3′OH ends of fragmented RNA, cDNA synthesis, Illumina NextSeq 500 sequencing. Greater than 10 million reads were obtained for each sample. Trimmed RNA-seq reads were mapped to the Pseudomonas aeruginosa UCBPP-PA14, complete genome (‘NC_008463’) using CLC Genomics Workbench and >99% of reads matched. The number of reads mapping to each gene was calculated and a matrix of read counts was generated. DESeq2 package was used to assess differential gene expression for all strains using the triplicate samples (18). All statistical analyses were performed in R version x64 4.0.2 (R Core Team (2020). R: a language and environment for statistical computing. R Foundation for Statistical Computing, Vienna, Austria. URL https://www.r-project.org/). Statistically significant differences in gene expression were assessed by the Wald test and adjusted for multiple comparisons using the Benjamini–Hochberg false-discovery rate correction. *P-*adjusted <0.05 was deemed significantly differentially expressed.

### ChIP-seq

All strains were grown overnight and sub-cultured in TSB. Strains were grown at 37°C to log phase the RNAP inhibitor Rifampicin was added to the polymerase control for 15 min. Samples were prepared like (19). Briefly, samples were cross-linked with 1% formaldehyde at 37°C for 20 min. Crosslinking was quenched with glycine (final concentration: 450 mM). Cells were pelleted, washed twice and frozen at −80°C. Pellets were suspended in 2 ml Immunoprecipitation (IP) buffer (50 mM HEPES−KOH pH 7.5, 150 mM NaCl, 1 mM EDTA, 1% Triton X-100, 0.1% sodium deoxycholate, 0.1% SDS) + 1× Complete Protease Inhibitor (CPI) (Sigma-Aldrich). DNA from these samples was sheared using the sonicator, Misonix Ultrasonic Processor S4000 (Boston Laboratory Equipment, USA), set to an Amplitude of 100 for a total of 10 min (pulsing at 30 s on, 30 s off), and the cell debris was removed. To ensure presence of correctly sized DNA (2–400 bp), 200 μl of each sample was de-crosslinked by incubation at 42°C/2 h, 65°C/6 h with 4 mg/ml Pronase (Sigma-Aldrich). These samples were purified using QIAquick PCR Purification kit (Qiagen) and analysed by gel electrophoresis. A 100 μl aliquot of the supernatant was removed and stored at −20°C to act as the ‘input’ sample, which would serve as the background control for ChIP-seq. The remainder of each sample was split into 2; to one half the antibody (Anti-Flag or Anti-RpoB) was added, the other was used as a negative control. Both were incubated at 4°C overnight on a rotating wheel. Sheep anti-Mouse IgG Dynabeads (Invitrogen, UK) (50 μl/sample) were washed twice with Phosphate Buffered Saline (PBS) and twice with IP buffer. Washed beads were saturated in IP + 1× CPI, with 1mg/ml BSA and incubated at 4°C overnight on the rotating wheel. The following morning, the beads were separated using a magnetic rack, and resuspended in IP + CPI (50 μl/sample). 50 μl of beads were added to each sample, which were then incubated at 4°C for 2 h. Beads were recovered using the magnetic rack, and washed twice in IP buffer, and once each in IP Buffer + NaCl (500 mM), Wash III (10 mM Tris pH 8, 250 mM LiCl, 1 mM EDTA, 0.5% Nonidet-P40, 0.5% sodium deoxycholate), Tris–EDTA buffer, pH 7.5 (10 mM Tris pH 7.5,1 mM EDTA). Beads were incubated in 100 μl elution buffer for 40 min at 65°C, shaking. The supernatant (containing the eluted nucleo-protein complex) was de-crosslinked as described above. DNA was purified using QIAGEN Minielute PCR Purification kit (Qiagen) and eluted in 15 μl MilliQ H_2_O. Qubit high sensitivity dsDNA assay (Life Technologies) was used to quantify the DNA. Libraries of ChIP-purified DNA were labelled using the TruSeq DNA sample preparation kit LT (Illumina) according to the manufacturer's instructions with the following modifications. Ten nanograms of ChIP-purified DNA was used to construct each library. An additional 5-cycle PCR was added before size selection of libraries to improve yields (PCR completed as described in amplification of libraries in the kit with PCR primers provided), also an additional gel extraction step was added following final PCR amplification to remove excess primer dimers. PCR amplification for ChIP libraries was completed using KOD Hotstart DNA Polymerase (Merck). The fragment size was confirmed using the high sensitivity DNA analysis kit on a 2100 Bioanalyser (Agilent). DNA libraries were multiplexed and sequenced using an Illumina NextSeq 500. Reads were mapped to UCBPP-PA14, complete genome (‘NC_008463’). Peaks were called against the input sample and reads were visualized and screenshots taken using Integrative Genome Viewer 2.8.0.

### Biofilm assay

Crystal violet 96-well biofilm assays were used to assess the impact of gene deletions upon biofilm formation similarly to that published previously (20). Assays were performed using 200 μl LB broth per well, in 96-well Falcon plates (353075), 24 h, 37°C static, using a randomized format to exclude edge bias. Sterile media replicates were negative controls. Biofilms staining using crystal violet were left for 15 minutes before washing and plate submerging in sterile purified water. Ethanol was added and left for 15 min to solubilise crystal violet, before shaking on a Bio-Rad plate shaker. Absorbances were read at 600 nm using a FLUOstar Omega plate reader.

### Swimming motility assay

Swimming motility was evaluated using tryptone agar plates (0.5% (w/v) NaCl, 1% (w/v) tryptone, 0.4% (w/v) agar) as previously described (21) leaving for a 20 minute hood drying period. Bacteria were inoculated, using a metal probe, from 200 μl aliquots of overnight cultures grown in LB broth (1% (w/v) NaCl, 0.5 (w/v) yeast extract, 1 (w/v) Tryptone). Plates were incubated in darkness overnight at 37°C for 24 h. Images of plates were taken and analysed using ImageJ (22) to quantify swimming by measuring area migrated over 24 h.

### Beta-galactosidase assay

Beta-galactosidase assay was performed as previously described (23). Briefly, bacterial cells were grown overnight in 5 ml TSB in falcon tubes, diluted to 0.1 and grown to mid log phase in 50 ml flasks. Approximately 1 OD unit was harvested and pelleted. Cells were resuspended in Z-buffer (Na_2_HPO_4_ 0.06M, NaH_2_PO_4_ 0.04M, KCl 0.01M, MgSO_4_ 0.001M and β-mercaptoethanol 0.05M). Cells were permeabilised with 50 μl 0.1% SDS, 100 μl chloroform and vortexing. After phase separation, lysate was added to a microtitre plate and Ortho-Nitrophenyl-β-galactoside at 4 mg/ml was added and the time noted. The plate was incubated at 28°C and monitored for colour development. Stop solution (Na_2_CO_3_ 1M) was added, time recorded and the plate was read at 405 and 540 nm prior to Miller units being calculated.

### T6SS competition assays

Competition assays were performed as we previously established with Top10 pRL662-*gfp* as the prey strain (3,24). Briefly overnight cultures were mixed 1:1 and spotted on LB agar plates for 5 h. Spots were recovered, resuspended, serially diluted and spotted on to LB, LB X-gal, LB gentamycin and/or PIA plates to enable colony counts.

### Secretion assays

Secretion assays were conducted as previously described (3) with minor modifications. Briefly, strains were inoculated into 25 ml of TSB at OD_600nm_ 0.1 and grown at 25°C shaking forhr 8 h. Spent media was cleared of cells by four rounds of centrifugation at 4000 g at 4°C, taking the uppermost supernatant each time. Proteins were precipitated with 10% trichloroacetic acid supplemented with 0.03% sodium deoxycholate overnight at 4°C on ice.

### Western blot analysis

Samples and Kaleidoscope Prestained Standard (Bio-Rad) were loaded and resolved in 8% (VgrGs, RpoB), 12% (RpoB, Myc, Flag) or 15% (Hcps and TssBs) gels using the Mini-PROTEAN system (Bio-Rad) by electrophoresis. Proteins were transferred to 0.22 μm nitrocellulose membranes (GE Healthcare). Membranes were blocked in Tris-buffered saline pH 8 with 0.1% Tween-20 (TBST) with 5% milk (Sigma) prior to incubation with antibodies. Monoclonal antibodies were used at the following dilutions: anti-RNA polymerase (Biolegend) at 1:5000, anti-Myc (Abcam) at 1:1000, anti-Flag-M2 (Sigma) at 1:1000. Polyclonal primary antibodies were used at a dilution of 1:1000 including; anti-Hcp1 (25), anti-Hcp2 (26), anti-TssB1 (27), anti-TssB2 (26), anti-VgrG2a and anti-VgrG2b (28), anti-VgrG4b (3) and anti-Hcp3 antibodies (3). Blots were washed with TBST prior to incubation with HRP-conjugated secondary antibodies (Sigma) mouse (for monoclonal antibodies) or rabbit (for polyclonal antibodies) at a dilution of 1:5000. Signals were detected using the Novex ECL HRP Chemiluminescent substrate (ThermoFisher) or the Luminata Forte Western HRP substrate (Millipore) using a LAS-3000 Fuji Imager or BioRad ChemiDoc XRS+. ECL detection and a white light image were taken separately. Adobe Photoshop was used to adjust the brightness/contrast of each blot uniformly prior to overlaying using the multiply tool and merge layer functions.

### Bioinformatics and statistical analysis

DNA sequences were retrieved from the Pseudomonas Genome Database (www.pseudomonas.com) or NCBI (www.ncbi.nlm.nih.gov) (29,30). DNA and amino acid sequence searches were executed using SMART (SMART: Change mode (embl-heidelberg.de)), InterPsoScan (About - InterPro (ebi.ac.uk)), Pfam (Pfam: Home page (xfam.org)), CDD (Conserved Domains Database (CDD) and Resources (nih.gov)), BLAST (BLAST: Basic Local Alignment Search Tool (nih.gov)) and Phyre2 (PHYRE2 Protein Fold Recognition Server (ic.ac.uk)) (31). Binding motifs, alignments, operons and promoter regions were investigated using, the Pseudomonas Genome Database (30), IGV (IGV: Integrative Genomics Viewer), Clustal (Clustal Omega < Multiple Sequence Alignment < EMBL-EBI), Mfold (17), FUZZNUC (EMBOSS), BPROM and FGENEB (www.softberry.com). Statistical analysis is detailed further in the figure legends but was performed using GraphPad Prism as indicated with the exception of the RNA-seq/ChIP-seq analysis which was performed in R.

## RESULTS

### RsmA is a global negative regulator of the core T6SS genes and associated *vgrG* islands

**Figure 1.**
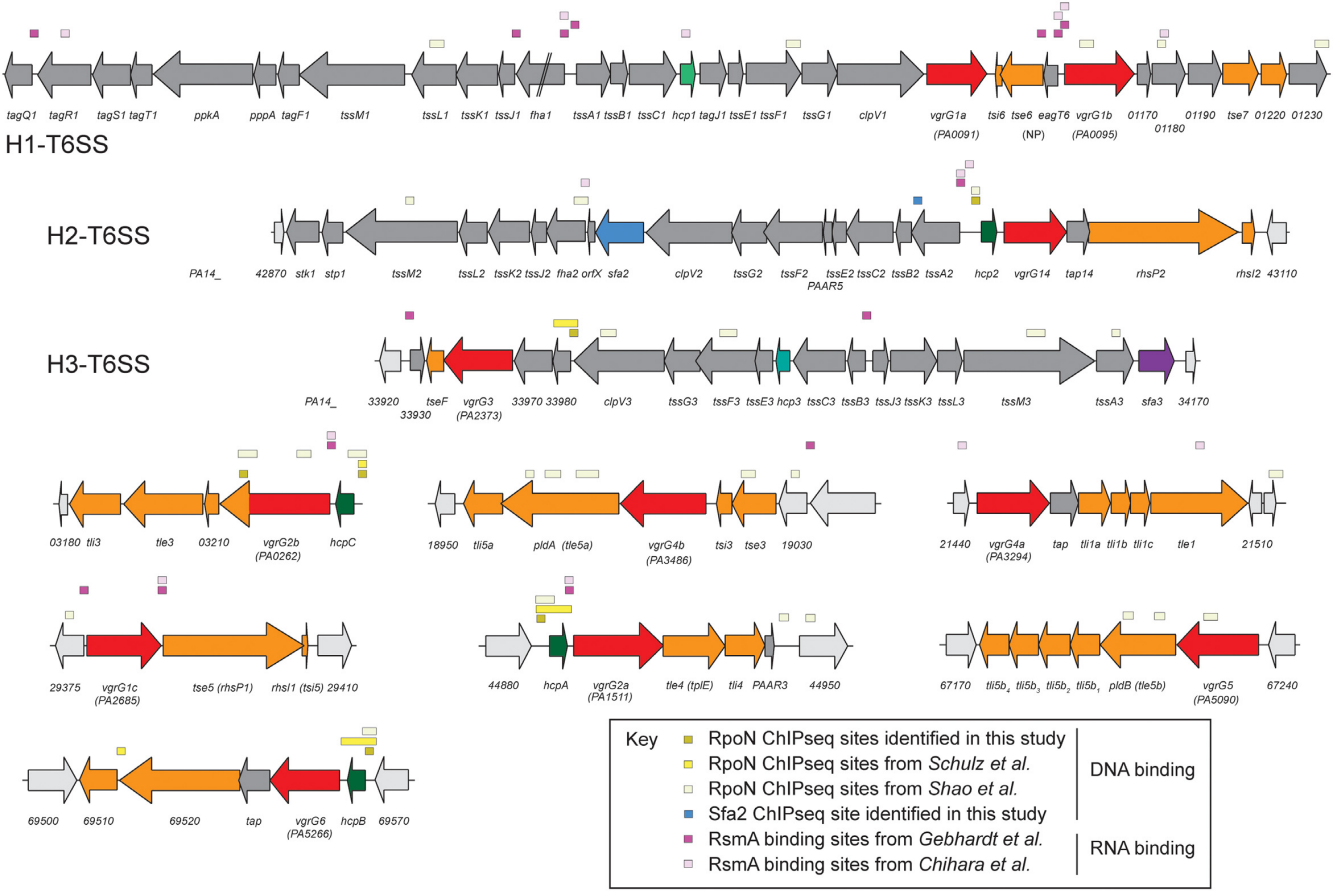
Schematic of T6SS clusters and *vgrG* operons in PA14, including the binding sites for RpoN and RsmA. T6SS associated genes in dark grey, unknown in light grey, *vgrG* genes in red, *hcp* genes in green, known or putative toxin and immunity proteins in orange, *sfa2* in blue and *sfa3* in purple. Binding motifs from this study and previously published work (13,34,35,38) are indicated above the genes and colour coded as presented in the key.

The regulatory network controlling expression of T6SS genes in *P. aeruginosa* has been widely studied and involves multiple branches that impact positively or negatively upon expression of the various T6SS core genes or all the associated *vgrG* gene islands (Figure 1). Among all described regulators it has been proposed that RsmA is the most global of all with a negative impact on the expression of all three main T6SS clusters, namely H1-, H2- and H3-T6SS (3). Here, we performed a comprehensive RNA-seq analysis of the differential expression between PA14*rsmA* and PA14 which showed that extensive de-repression occurs with 644 genes differentially expressed with a *P* value of 0.05 (Supplementary Figure S1, Table 1, Supplementary Table S4). Of those 504 (8.16%) of the genes were altered ≥1.5-fold with 363 genes being upregulated in PA14*rsmA* (Table 2, Supplementary Table S4). The RNA-seq results were in concordance with the previously published microarray, RNA-seq, ChIPPAR-seq or UV ChIP-seq data showing that greater than 500 genes are altered upon deletion of *rsmA* in PAO1 or PAK (32–35). Remarkably, we observed significantly altered expression of 92 (82.1%) of the 112 known T6SSs genes in PA14 (Supplementary Figure S1A, Table S4). A clear visual demonstration of this is in Supplementary Figure S1B, that shows increased expression for 6/6 *hcp* genes (3.28–16.46-fold) as well as 10/11 of the *vgrG* genes (2.20–12.56-fold) but *vgrG3* was not impacted (Supplementary Figure S1B, Table S4). Of the 20 genes not significantly altered, 12 of these are in the H3-T6SS central cluster. Six out of the top 15 most significant genes belong to the T6SSs (Supplementary Figure S1C and Table S4). The other top seven genes include: the *magB-F* (PA14_58230–70) operon known to be regulated by RsmA that encodes a periplasmic complex shown to inhibit neutrophil elastase (36), a putative contact dependent inhibition subunit A protein (PA14_00510) (37), a putative Zn-dependent M48 family metallopeptidase containing protein with a potential lipoprotein signal peptide (PA14_03610), a predicted ATPase involved in DNA repair cell division and chromosome partitioning (PA14_16190), and a putative WG repeat-containing protein with a lipoprotein signal peptide (PA14_16330) (Supplementary Figure S1C and Table S4). RsmA mediates its global control by directly repressing or enhancing mRNA stability and translation through direct binding of mRNAs, but also indirectly through regulating other transcriptional and post-transcriptional regulators. Mapping of published RsmA bound mRNAs from ChIP-seq experiments from Chihara *et al.* and Gebhardt *et al.* identified multiple mRNA targets encoded by all three T6SS central clusters and most *vgrG* islands demonstrating the coverage that RsmA has on T6SS control (Figure 1) (34,35). However, as not all T6SS operons or genes have corresponding mRNAs that are directly bound by RsmA but have altered gene expression profiles, they are likely indirectly controlled. Indeed, previous studies have shown that RsmA alters translation of specific regulatory factors that lead to transcriptional changes, broadening the impact of RsmA (32–35). In summary, this expands our previous work showing RsmA controls components of all three T6SSs (3) and highlights the global control of RsmA over the T6SS genes occurring both directly and indirectly.

**Table 1.** Summary of RNA-seq results 0.05

	*rsmA* vs PA14	*rsmArpoN* vs *rsmA*	*rsmAsfa2* vs *rsmA*	*rsmAsfa3* vs *rsmA*	*rsmArpoN* vs PA14
LFC > 0 (up)	442 (7.16%)	1092 (17.68%)	10 (0.16%)	4 (0.07%)	1298 (21.01%)
LFC < 0 (down)	202 (3.27%)	1100 (17.81%)	41 (0.66%)	7 (0.11%)	1271 (20.57%)
Total	644 (10.43%)	2192 (35.49%)	51 (0.83%)	11 (0.18%)	2569 (41.59%)

Note: LFC = log_2_ fold change.

**Table 2.** Summary of RNA-seq results 0.05 and >< Log_2_ 0.58 (1.5 Fold)

	*rsmA* vs PA14	*rsmArpoN* vs *rsmA*	*rsmAsfa2* vs *rsmA*	*rsmAsfa*3 vs *rsmA*	*rsmArpoN* vs PA14
LFC > 0.58 (up)	363 (5.88%)	756 (12.24%)	10 (0.16%)	4 (0.07%)	997 (16.14%)
LFC < 0.58 (down)	141 (2.28%)	798 (12.92%)	39 (0.63%)	6 (0.1%)	918 (14.86%)
Total	504 (8.16%)	1554 (25.16%)	49 (0.79%)	10 (0.16%)	1915 (31%)

Note: LFC = log_2_ fold change.

### RpoN-dependent expression of *P. aeruginosa* T6SS genes

**Figure 2.**
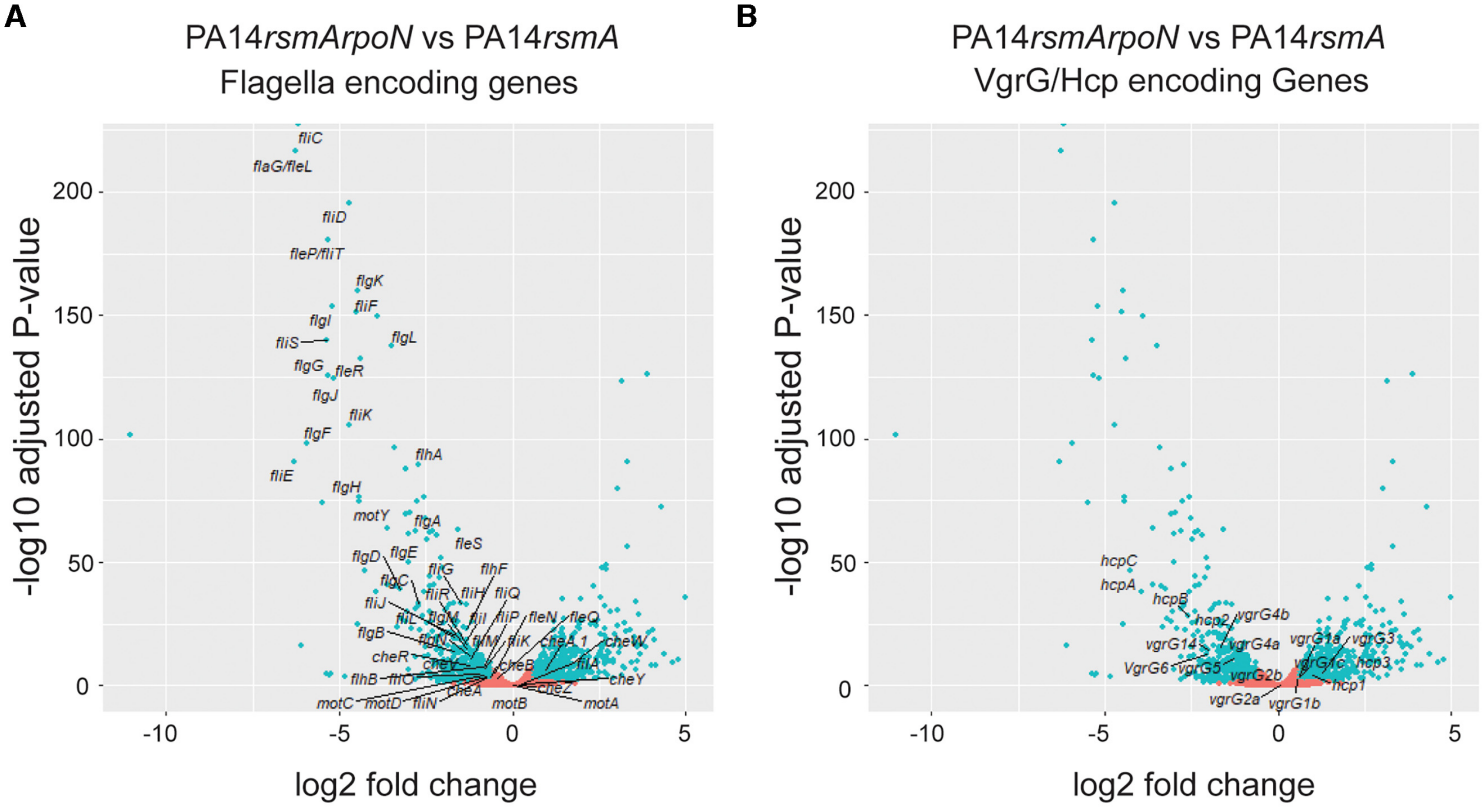
RpoN-dependent expression of *P. aeruginosa* T6SS genes. RpoN is a positive regulator of (**A**) Flagella regulon and (**B**) H2-T6SS (*vgrG14/4a/4b/5, hcp2/A/B/C*) but a negative regulator of the H1-(*vgrG1a/c, hcp1*) and H3-T6SS (*vgrG3, hcp3*). Volcano plots of differentially expressed genes with colours indicating each gene's absolute log2 (fold change): orange ≤0.58; and blue >0.58 (1.5-fold) with a *P-*adjusted <0.05 (*n* = 3). Significance was determined by a Wald test and adjusted for multiple comparisons using the Benjamini–Hochberg false-discovery rate correction using DESeq2.

Two of the core T6SS clusters, H2- and H3-T6SS, encode SFA proteins which suggests that RpoN could be involved in controlling the expression of these gene clusters (Figure 1). We thus analysed the effect of a *rpoN* deletion in the T6SS-active *rsmA* background. Our global RNA-seq approach revealed 2192 genes were differentially expressed and of those 1554 genes (25.16%) were ≥1.5 fold altered expression when comparing PA14*rsmArpoN*/PA14*rsmA* (Tables 1 and 2 and Supplementary Table S5). An almost equal split was observed with 756 (12.24%) genes showing increased expression, whilst 798 (12.92%) genes were repressed (Tables 1 and 2). We observed extensive deregulation of genes previously identified as part of the RpoN regulon (6,7,13,38) with 12 of the top 15 highest genes encoding components associated with flagella biogenesis (Figure 2A, Supplementary Figure S2A, Table S5). Inspection of the other three genes showed: *gcbA* (PA14_64050) encoding a diguanylate cyclase which helps to facilitate biofilm dispersion and regulate flagellar motility (39,40), a predicted cysteine hydrolase with a solved crystal structure encoded by PA14_48760 (41) and PA14_07430 which encodes ImpA a T2SS secreted metallopeptidase involved in preventing the correct functioning of neutrophils and macrophages (42,43).

Further analysis showed that 48 of 55 (87.27%) of genes associated with the flagella system were significantly altered ≥1.5-fold (7,44) in our *rpoN* mutant, which is reflected not only by the loss of flagella-based motility (Figure 2A and Supplementary Figure S3) (7) but also by reduced biofilm formation (Supplementary Figure S4) (20). With respect to the T6SS we observed a significantly altered expression ≥1.5-fold for 62/112 (55.4%) of the T6SS genes (Supplementary Figure S2B). However as opposed to RsmA, there is a greater variety in the impact of RpoN. For example, expression of genes encoding components of the H1-T6SS were modestly elevated (1.53–1.97-fold), those encoding components of the H2-T6SS system were repressed (−1.55 to −4.89 fold), whilst the H3-T6SS was activated (2–7.42-fold) in the PA14*rsmArpoN* background compared to PA14*rsmA* (Supplementary Table S5 and Figure S2B). Looking further into *hcp* genes or *vgrG* islands (Figure 1), this trend is confirmed since those associated with the H1-T6SS, e.g. *vgrG1abc* and *hcp1*, are unchanged or activated, those associated with H2-T6SS, e.g. *vgrG2a, vgrG2b*, *vgrG4a, vgrG4b*, *vgrG5*, *vgrG6*, *vgrG14* and *hcp2ABC*, are all repressed and those linked with the H3-T6SS, e.g. *vgrG3* and *hcp3*, are activated (Figure 2B & Supplementary Table S5). Overall, these data support a global RpoN control on T6SS genes, including the H1-T6SS cluster that does not encode an SFA.

### Assembly and activity of the T6SS in a *rpoN* background

**Figure 3.**
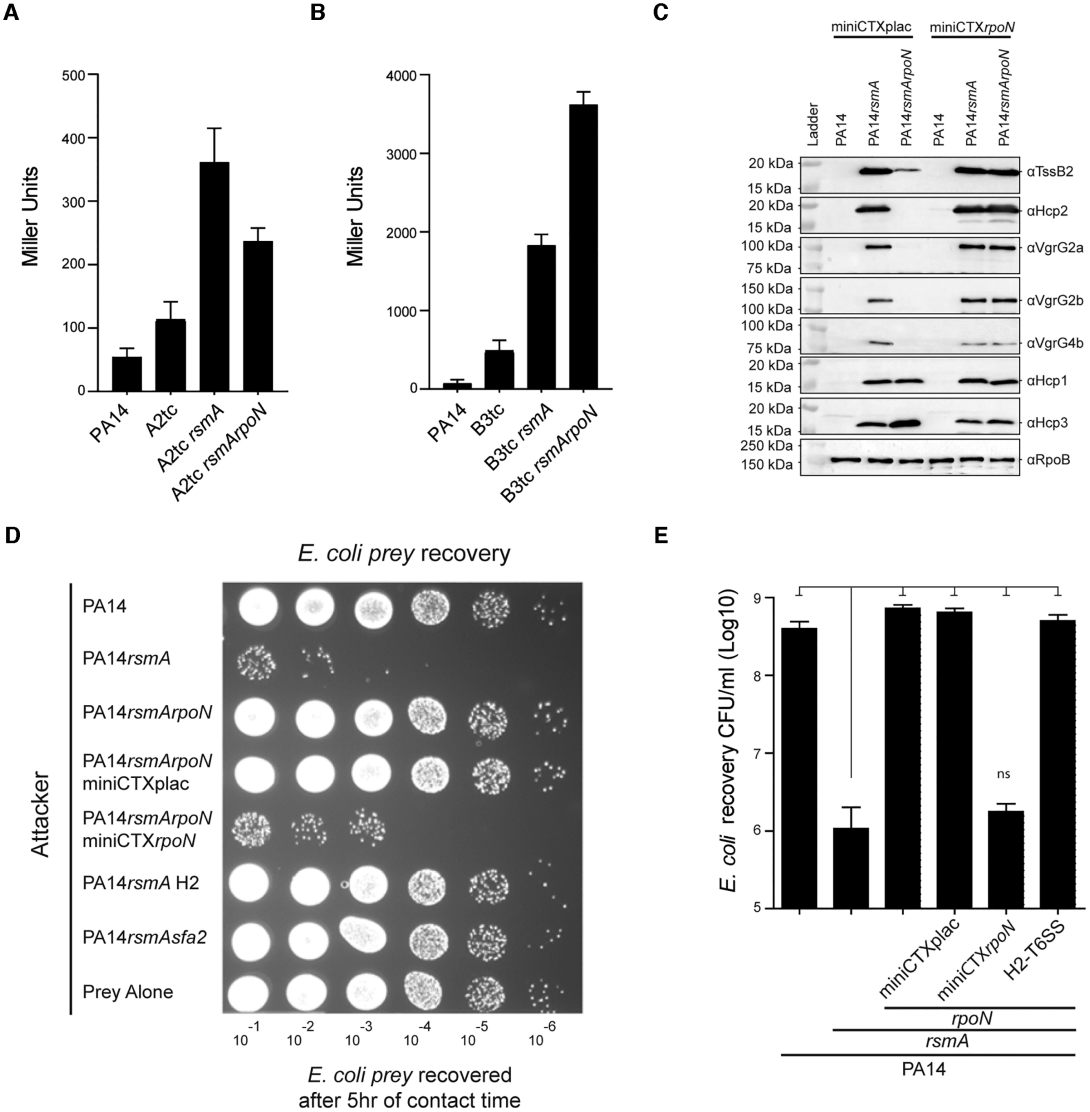
Assembly and activity of the T6SS in a *rpoN* background. Deletion of *rpoN* abrogates expression and killing via the H2-T6SS. (**A**) Beta-galactosidase assay confirms *rpoN* deletion reduces expression of H2-T6SS components [*tssA2* transcriptional fusion (A2tc)]. (**B**) Beta-galactosidase assay confirms *rpoN* deletion enhances expression of H3-T6SS components [*tssB3* transcriptional fusion (B3tc)]. Graphs represents mean + SD (*n*= 3, ANOVA, Tukey's multiple comparison Test, *P* < 0.01). (**C**) Deletion of *rpoN* reduces or abolishes expression of the H2-T6SS core and orphan components but results in increased expression of the H3-T6SS component Hcp3. Western blot analysis of whole cell lysate of mutant strains using specific antibodies against H2-T6SS components (Hcp2, TssB2, VgrG2a, VgrG2b and VgrG4b), H1-T6SS (Hcp1), H3-T6SS (Hcp3), or RpoB as a control. Complementation of *rpoN* using miniCTX*rpoN* restores production of H2-T6SS components and decreases Hcp3 level. (**D**) RpoN is essential for H2-T6SS bacterial killing. Recovered *E. coli* GFP-tagged prey selected on Gm plates after co-incubation of the 1:1 bacterial mix and serial dilution. Deletion of *rsmA* is required for H2-T6SS killing of *E. coli*, as the prey is recovered significantly less when co-incubated with PA14*rsmA* as compared to PA14. Deletion of *rpoN* in PA14*rsmA* abolishes killing. Complementation restores killing. (**E**) Quantification of bacterial killing assay using colony counts in C. Graph represent mean + SD (*n* = 3, ANOVA, Dunnett's posttest *P* < 0.001).

To validate the above observations, we used cognate H2- (*tssA2*) and H3-T6SS (*tssB3*)-*lacZ*-reporter fusions and engineered a set of specific *P. aeruginosa* mutants. As expected, deletion of *rsmA* resulted in increased activity for both H2- and H3-T6SS (Figure 3AB). Subsequent deletion of *rpoN* resulted in decreased activity for the H2-T6SS reporter but elevated levels of activity for the H3-T6SS reporter confirming our RNA-seq results described above (Figure 3AB) and the antagonistic RpoN-dependent control on H2- and H3-T6SS genes.

We subsequently assessed if control on gene expression is reflected in protein production levels. Western blot analysis using specific antibodies confirmed increased production of components from all three T6SS in a *rsmA* mutant supporting our previous findings (3) (Figure 3C). Yet, deletion of *rpoN* in the *rsmA* background resulted in decreased level of the sheath component TssB2 as well as the H2-T6SS specific Hcp2 proteins (Figure 3C). Strikingly deletion of *rpoN* also abolished production of VgrG proteins encoded in H2-T6SS-associated *vgrG* islands, namely VgrG2a, VgrG2b and VgrG4b. We thus concluded that RpoN is required for the coordinated expression of core and tip complex components of the H2-T6SS. Finally, western blot analysis showed elevated levels of the H3-T6SS component Hcp3 in the PA14*rsmArpoN* strain (Figure 3C), thus supporting the H3-T6SS data retrieved from the RNA-seq experiment and from the *H3-T6SS-lacZ* reporter analysis (Figures 2B and 3B). In conclusion, RpoN has an antagonistic impact on H2- and H3-T6SS by playing an activating role on H2-T6SS but having a repressive impact on the H3-T6SS.

We have shown previously that most *vgrG* islands in *P. aeruginosa* encode putative antibacterial toxins and are usually associated with the H2-T6SS. Here, we assessed the phenotypic effect of a *rpoN* deletion by exploring the H2-T6SS-dependent bacterial killing using conditions we previously established (3). As shown before, killing of an *E. coli* prey is induced in a PA14*rsmA* mutant but introducing the *rpoN* mutation to generate a *rsmArpoN* double mutant results in loss of killing (Figure 3DE). Complementation of *rpoN* using a chromosomally integrated plasmid miniCTXplac vector harbouring *rpoN* fully restored killing (Figure 3DE). The bacterial killing was H2-T6SS-mediated as no killing was observed in a PA14*rsmA* H2-T6SS mutant (Figure 3DE).

Overall, RpoN modulates expression/activity of all three T6SS clusters by repressing H1- and H3-T6SS, but is an activator of the H2-T6SS core cluster and related orphan operons, all required for robust interbacterial killing, which is in marked contrast with what was previously reported (12).

### Role of Sfa2 in mediating T6SS expression

**Figure 4.**
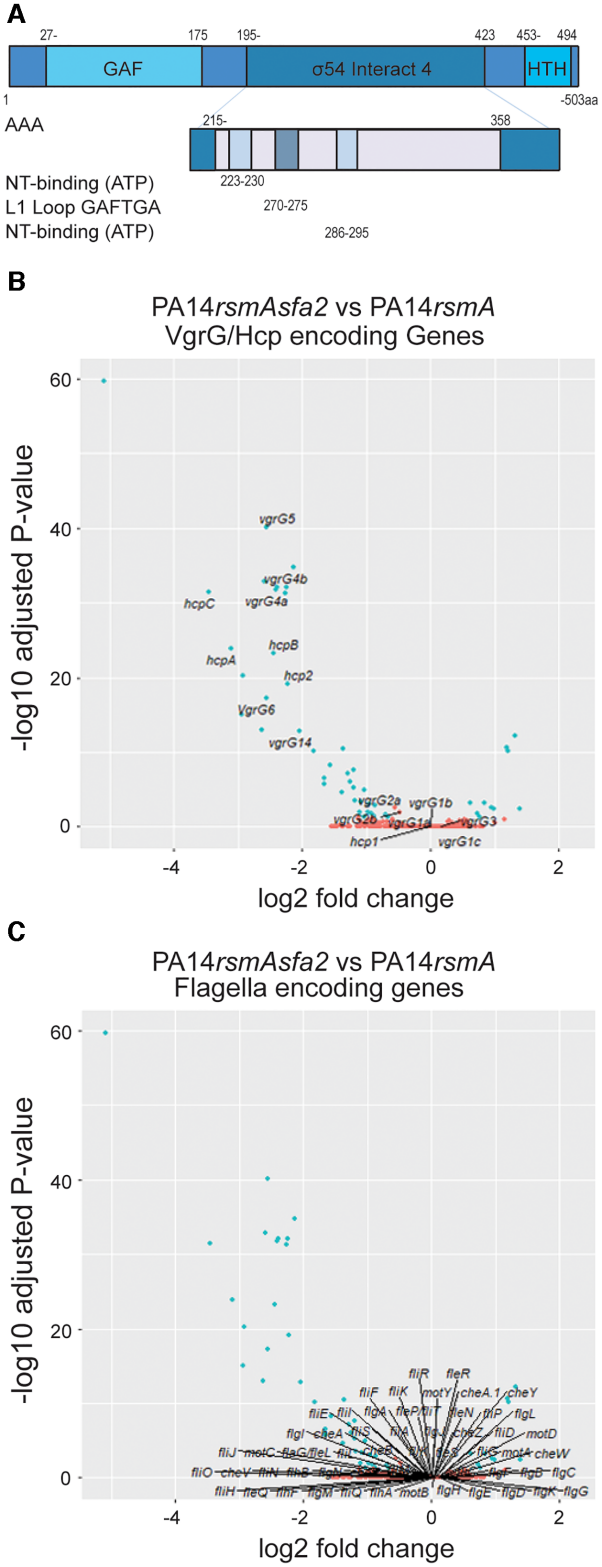
Sfa2-mediated control of the H2-T6SS. (**A**) Domain structure of Sfa2. (**B**) Sfa2 is a positive regulator of the H2-T6SS specifically *vgrG14/4a/4b/5, hcp2/A/B/C*. (**C**) Sfa2 has no significant effect on genes in the flagella regulon. Volcano plots of differentially expressed genes with colours indicating each gene's absolute log2 (fold change): orange ≤ 0.58; and blue > 0.58 (1.5 fold) with a *P-*adjusted < 0.05 (*n* = 3). Significance was determined by a Wald test with Benjamini–Hochberg correction.

The alternative sigma factor RpoN, generally acts in concert with an enhancer binding protein (EBP) or a sigma 54 activator (SFA) protein for activation and DNA promoter opening (45,46). Here, we have shown RpoN’s involvement in positively controlling H2-T6SS expression. Within the H2-T6SS cluster is a gene annotated *sfa2* that encodes for a protein containing an N-terminal GAF domain, Sigma 54 interaction domain and a helix-turn-helix DNA binding domain (Figures 1 and 4A).

Here we further investigated whether Sfa2 acts in concert with RpoN and is required for H2-T6SS expression. We used a global approach by performing RNA-seq analysis of a *sfa2* mutant (in the *rsmA* background). Our data revealed a significantly smaller regulon, as compared to RpoN, with 49 (0.79%) differentially expressed genes (Figure 4B, Tables 1 and 2) with no alteration to flagellar biogenesis genes (Figure 4C) which is an expected outcome since FleQ is present. Supporting this, flagella-based swimming motility and biofilm formation capacity were also not altered in the *sfa2* mutant in contrast to the *rpoN* mutant (Supplementary Figures S3 and S4). Yet, and most remarkably, the expression of 34 of the H2-T6SS genes was reduced/modulated (Figure 4B, Supplementary Figure S5A, Table S6). Of the top 15 most significant genes all are encoded in the H2-T6SS core cluster and H2-linked orphan operons (Supplementary Figure S5AB, Table S6). Sfa2 function appears to be very specific, as no genes were significantly altered that are linked with the H1- or H3-T6SS (Supplementary Figure S5A, Table S6), thus restricting and focusing the impact of RpoN on the H2-T6SS-related genes.

The impact of Sfa2 on the core H2-T6SS clusters might explain the results that were obtained in a previous study where we performed a transposon insertion screen for regulators of the H2-T6SS using a *tssA2-lacZ* promoter fusion as a readout (3). Notably, a transposon insertion into *tssG2* within the H2-T6SS cluster resulted in increased activity from the *tssA2* promoter that is located upstream of *tssG2* (Supplementary Figure S6AB). As the Himar1 Mariner C9 transposon used for mutagenesis contains outward facing promoters (47), activation of genes downstream this insertion would result in elevated levels of Sfa2. Sfa2 would then be free to bind and act in conjunction with RpoN to promote expression of itself and all the genes from the central H2-T6SS promoter region (Supplementary Figure S6B), which was what we observed. We thus conclude that Sfa2 is specifically required for production of the genes encoding the H2-T6SS.

### Role of Sfa2 in mediating T6SS activity

**Figure 5.**
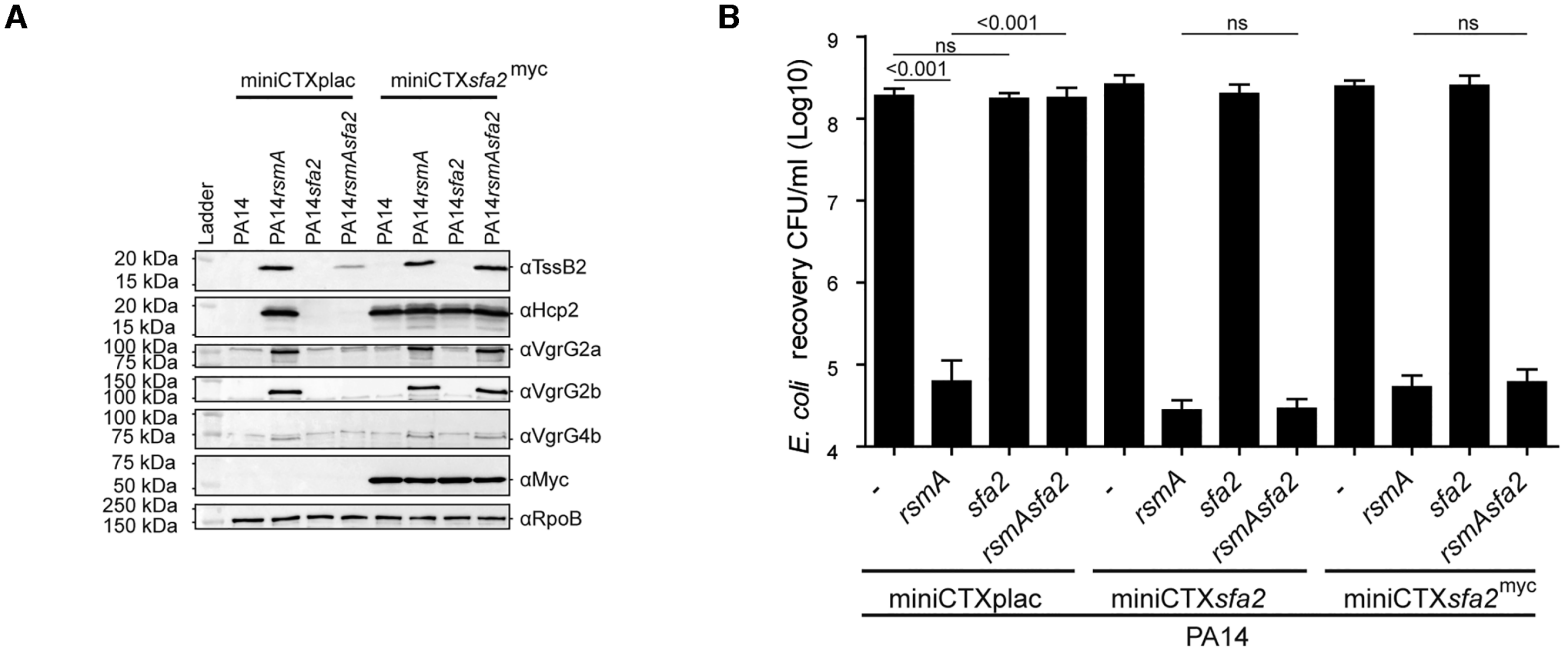
Assembly and activity of the T6SS in a *sfa2* background. Deletion of *sfa2* abrogates expression and killing by the H2-T6SS. (**A**) Deletion of *sfa2* reduces or abolishes production of H2-T6SS components. Western blot analysis of whole cell lysate of mutant strains using specific antibodies against H2-T6SS components (TssB2, Hcp2, VgrG2a, VgrG2b and VgrG4b), Myc epitope tag for Sfa2^myc^ or RpoB as a control. Complementation of *sfa2* using miniCTX*sfa2^myc^* restores production of these H2-T6SS components. (**B**) Sfa2 is required for H2-T6SS bacterial killing. Quantification of bacterial killing assay after incubation of *E. coli* and PA14 attackers. Deletion of *rsmA* enables H2-T6SS-mediated bacterial killing. The killing is lost in an *rsmA/rpoN* mutant but restored upon complementation with *rpoN*. Quantification is made using colony counts (See Supplementary Figure S8). Graph represent mean + SD; *n* = 3; statistical significance is indicated using one-way ANOVA with Dunnett's posttest *P* < 0.001.

Supporting our gene expression readouts, western blot analysis confirmed that deletion of *sfa2* reduces expression of the H2-T6SS sheath protein TssB2 and abolishes expression of the H2-T6SS Hcps (Figure 5A). Complementation with miniCTX*sfa2*^myc^ fully restored expression of these components and elevated expression of the Hcp2. To confirm the role of Sfa2 in other *P. aeruginosa* isolates we engineered mutants in another prototypical *P. aeruginosa* laboratory strain, PAO1. Deletion of *sfa2* in PAO1*rsmA* resulted in the complete loss of expression of Hcp2 (Supplementary Figure S7). Complementation of the *sfa2* mutant restored both Hcp2 expression and secretion (Supplementary Figure S7). Thus, Sfa2 is required for a functional H2-T6SS not only in PA14 but likely in most other *P. aeruginosa* isolates.

Our *sfa2* RNA-seq results showed decreased expression of multiple genes from the H2-T6SS core cluster and all H2-T6SS-associated *vgrG* islands (*vgrG2a*,*2b*,*4a*,*4b*,*5*,*6*,*14*) had reduced levels (−1.4 to −5.87 fold), whilst the H1-T6SS-associated genes, *vgrG1abc*, or H3-T6SS *vgrG3* gene were unaffected (−1.04–1.11-fold) (Figure 4 and Supplementary Table S6). To investigate the impact of deletion of *sfa2* on the production of these proteins we used specific antibodies against VgrG2a, VgrG2b and VgrG4b. We show that expression of these VgrG proteins is lost in the *rsmAsfa2* background and complementation of the *sfa2* mutant restores expression (Figure 5A). Thus, Sfa2 promotes expression of all H2-T6SS components in the core cluster and coordinates the expression of the orphan gene islands spread throughout the genome. This enables the expression of the widest range of H2-T6SS VgrG tips and their associated arsenal of antibacterial toxins for maximum functionality.

To ascertain the impact of the deletion of *sfa2* on toxins delivered by the VgrG tips and thus upon interbacterial killing we performed a competition assay. PA14*rsmA* effectively kills *E. coli* prey with a three-log reduction in prey recovery (Figure 5B, Supplementary Figure S8) but killing is lost in PA14*rsmAsfa2*. Complementation after the introduction of miniCTX*sfa2* or miniCTX*sfa2*^myc^ fully restores killing (Figure 5B). These results corroborate those observed with the *rpoN* mutant confirming that both RpoN and Sfa2 act in conjunction to control H2-T6SS killing.

### ChIP-seq analysis shows direct binding of RpoN and Sfa2 on T6SS promoter regions

**Figure 6.**
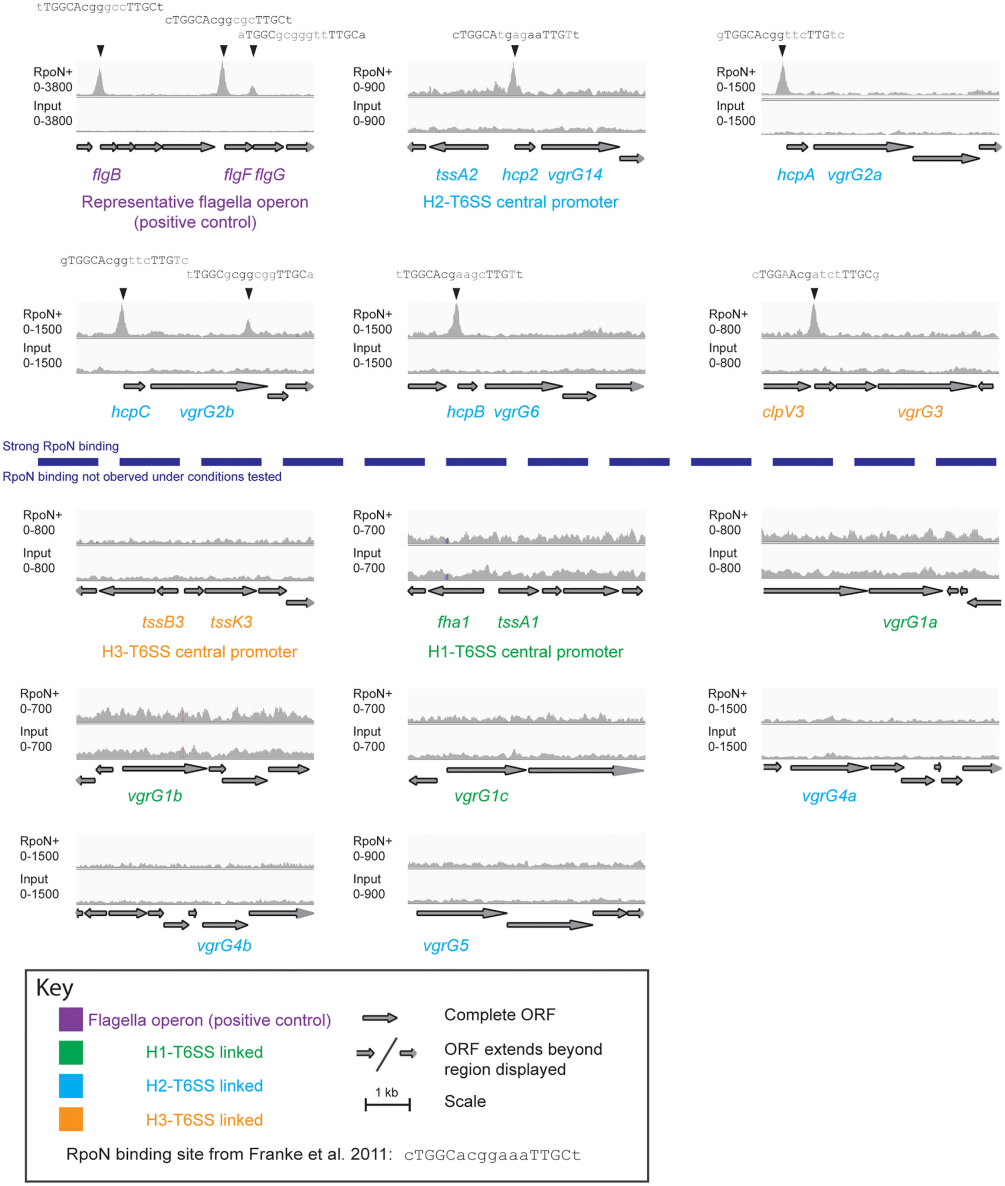
ChIP-seq reveals RpoN binding sites present in several H2-T6SS orphan gene clusters indicating direct control by RpoN. The blue dashed line separates those with clear RpoN binding and those not bound. The top track in each Integrative Genome Viewer image shows the RpoN ChiP enriched (RpoN+) binding profiles in proximity to genes of interest. A track with the input DNA (input) is shown as a negative control. The key indicates which system the displayed genes are associated with. A region of the flagella operon covering *flgB* to *flgH* with clear RpoN binding is included as a positive control. Predicted RpoN binding motifs identified in the centre of the ChIP enriched peaks are indicated with an arrow. Capitalisation in binding motif indicates the most highly conserved residues identified in Francke *et al.* 2011. Black bases indicate optimal residue compared to previously identified binding motif and grey bases indicate divergence.

To investigate how RpoN/Sfa2 controls H2-T6SS expression, we performed ChIP-seq using chromosomally-encoded Flag-tagged RpoN and Sfa2. For RpoN, binding was observed upstream of several H2-T6SS genes, including *tssA2*, *hcp2*, *vgrG14*, *vgrG2a*, *vgrG2b, vgrG6*, but also the H3-T6SS-associated *vgrG3* (Figures 1 and 6, Supplementary Table S7), confirming that RpoN directly regulates multiple T6SS clusters. Inspection of promoter regions revealed clear RpoN binding sites upstream of *tssA2*, *hcp2*, *vgrG14*, *vgrG2a*, *vgrG2b, vgrG6* and *vgrG3* (Figure 6). Notably, for *vgrG2a*, *vgrG2b*, *vgrG6* and *vgrG14* (Figure 6), the promoter regions are upstream of *hcp2* homologues (*hcp2ABC*) and all display a conserved sequence that corresponds to the ribosomal binding site, a RpoN binding site and conserved or inverted repeats which may be DNA transcription factor binding targets (Supplementary Figure S9). The presence of a RpoN binding site in the H3-T6SS cluster downstream of *clpV3* and upstream of *vgrG3* can be correlated with a direct inhibitory effect (Figures 3 and 6) and RpoN can play a repressive role through direct binding (48). In this scenario, RpoN binding may block the progression of RNA polymerase and the lack of a core H3-T6SS component such as VgrG3 would prevent the assembly of a functional H3-T6SS apparatus. We did not observe RpoN binding to the H1-T6SS central cluster or to four of the *vgrG* operons (Figure 1). However, mapping of ChIP-seq data from Schulz *et al.* and Shao *et al.* extended our analysis and suggests that RpoN may have a more substantial role in control by binding and modulating the H1-T6SS under different environmental conditions including growth in LB broth for both PAO1 and PA14 (Figure 1) (13,38).

Overall, our ChIP-seq approach is validated by the fact that RpoN is shown to bind to its well-known targets in our data set, including the top hit *glnA* (encoding glutamine synthetase involved with nitrogen metabolism) (49) (Supplementary Table S7). Binding was also observed upstream of *rpoN* itself as it is known to regulate its own expression (50) and clear congruence can be observed with the data from Shao *et al.* and Schulz *et al.* (13,38). Finally, binding in close proximity to known flagella motility genes including 6 in the top 20 (Supplementary Table S6, Table S7), and binding upstream of *flgB*, *flgF* and *flgG* as shown in Figure 6, support the data.

We also performed ChIP-seq analysis with Sfa2^Flag^ but this did not return as many results as with RpoN. Overall, the sample appears to come with a high level of genomic DNA background indicating poor enrichment. Yet, a site of enrichment can be seen overlapping the *tssA2* gene in proximity of the promoter region of the divergently facing *tssA2* and *hcp2* genes (Supplementary Figure S10). Due to the lack of expression at the mRNA or protein level in the *sfa2* or *rpoN* mutant, this binding of Sfa2 in proximity to RpoN and RNA polymerase peaks indicates that enhancer-dependent transcription is occurring (Supplementary Figure S10). However, this Sfa2 binding site is 2132 bp upstream of the RpoN binding site, within the H2-T6SS promoter region between the *tssA2* and *hcp2* genes. SFA or EBP typically bind closer to the genes they control but EBP sites can be more than 1 kb away from the RpoN binding site and still be functional (51,52). No other clear Sfa2 binding sites were observed upstream of any other clusters, suggesting this is the highest affinity site. Interestingly, a putative integration host factor (IHF) binding site could be identified between the Sfa2 and RpoN sites (Supplementary Figure S10) (53). IHF promotes DNA bending which facilitates the physical interaction between EBPs and promoter-bound RpoN–RNA polymerase required for activation. Such a scenario is documented for the Sfa2 homologue, VasH, in *Vibrio cholerae*, and provides further support for the direct role of Sfa2 and RpoN in control of the *P. aeruginosa* H2-T6SS central cluster (54).

### Specific role for Sfa2 and Sfa3 in coordinating T6SS activity

**Figure 7.**
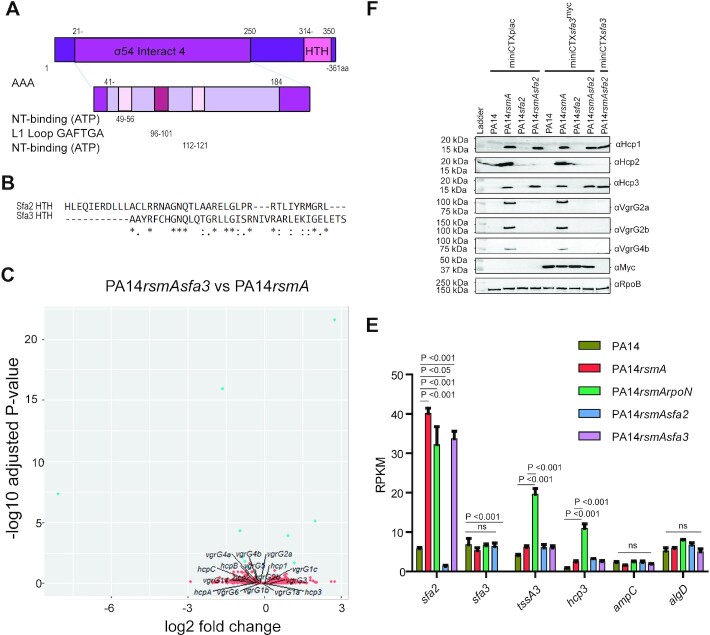
Sfa3 is an EBP but does not mediate control of the *P. aeruginosa* T6SSs. (**A**) Domain structure of Sfa3. (**B**) Alignment of HTH domains of Sfa2 and Sfa3 identifies differences suggesting binding specificities. (**C**) Sfa3 does not control *vgrG* or *hcp* genes under the experimental conditions tested. Volcano plots of differentially expressed genes with colours indicating each gene's absolute log_2_(fold change): orange ≤0.58; and blue >0.58 (1.5-fold) with a *P-*adjusted <0.05 (*n* = 3). Significance was determined by a Wald test with Benjamini–Hochberg correction. (**D**) *sfa3* cannot complement deletion of *sfa2* to restore production of H2-T6SS components. Western blot analysis of a panel of T6SS proteins. Expression of H2-T6SS components (Hcp2, VgrG2a, VgrG2b and VgrG4b) is lost in a *rsmAsfa2* mutant. Complementation of PA14*rsmAsfa2* with miniCTX*sfa3^myc^* or miniCTX*sfa3* does not restore expression of these H2-T6SS components nor elevate levels of the H1-T6SS component, Hcp1, or the H3-T6SS component, Hcp3. (**E**) *sfa3* is expressed at a low level and does not increase in a *rsmA* or *rsmArpoN* mutant unlike *sfa2*, *tssB3* or *hcp3*. Analysis of RNA-seq results using Reads per kilobase of exon model per million reads (RPKM), two-way ANOVA with repeat measurements and Bonferroni post-tests (*n* = 3). *ampC* and *algD* are included as housekeeping gene controls that are expressed in similar abundance to the genes of interest.

As previously reported, there are two Sfa proteins encoded within the H2- and H3-T6SS gene clusters (Figure 1). Alignment of Sfa2 and Sfa3 shows a relatively high level of identify (38%) over the central region of the proteins (Supplementary Figure S11A). However, this drops to 25% over the full length of the proteins due to differences in the N- and C-terminal regions. Sfa2 has an additional N-terminal 174 amino acids encoding a putative GAF domain (Figures 4A and 7A, Supplementary Figure S11A). GAF domains with a broad range of functions are present in ∼10% of EBPs and usually serve as sensory input sites for regulatory functions typically with inhibitory roles (6). This N-terminal GAF domain places Sfa2 in the type 1b EBP and Sfa3 in the type 1c EBP following the nomenclature outlined in Francke *et al.* (6). However, both Sfa2 and Sfa3 have a modular structure with the hallmarks of EBPs with clear RpoN interaction domains; two AAA ATPase P-loop motifs, a conserved amino acid stretch of GAFTGA that mediates interaction with RpoN and finally a helix-turn-helix domain for DNA interaction (Figures 4A and 7A) (55). Differences in the HTH motifs in the C-terminal regions suggest Sfa2 and Sfa3 bind to different specific DNA sequences, which would occur in proximity to RpoN binding sites, and this difference might confer specificity (Figure 7B).

We investigated whether Sfa3 could impact the expression of a specific subset of genes (Figure 7C). As was the case for the *sfa2* mutant and in contrast to the *rpoN* mutant, deletion of *sfa3* did not alter flagella associated genes, bacterial swimming motility or biofilm formation (Supplementary Figures S3 and S4, S11B). RNA-seq analysis of *sfa3rsmA* mutant compared to *rsmA* only identified 10 genes or 0.16% and only one (*tli5a*) was T6SS associated (Supplementary Figure S11CD). This gene is part of the *vgrG4b*/*pldA* cluster, with VgrG4b and PldA secreted *via* the H2-T6SS (56) and not the H3-T6SS (Figures 1 and 7C, Supplementary Figure S11C, Table S8). This could indicate cross-regulation between the H2- and H3-T6SS and to probe this, miniCTX*sfa3* was used to complement deletion of *sfa2* to test the functional specificity. However, *sfa3* was unable to restore expression of any of the H2-T6SS components and thus cannot compensate for the lack of Sfa2 (Figure 7D). In addition, the lack of VgrG4b expression in a *sfa2* mutant complemented with *sfa3* does not favour this hypothesis of cross regulation of the systems via the EBPs or enhanced expression of the immunity gene *tli5a* that is encoded in the *vgrG4b* orphan cluster (Figures 1 and 7D). Instead, such a small number of genes (0.16%) may suggest they are false positives (Supplementary Table S8). The lack of clear impact of Sfa3 on the H3-T6SS may suggest it is not highly expressed under our experimental conditions. Indeed, the levels of *sfa3* expression in a *rsmA* or in a *rsmArpoN* mutant are not increased in contrast with the high level of *sfa2* expression in these same backgrounds (Figure 7E). However, the level of other H3-T6SS genes such as *tssA3*, *tssB3* and *hcp3* is elevated in the *rsmA/rpoN* background suggesting Sfa3 is not essential for the RpoN-dependent control on H3-T6SS genes but could contribute to further elevated expression. It should be noted that in contrast to *sfa2*, the *sfa3* gene is not embedded in the H3-T6SS cluster but at the end of it and both BPROM and FGENEB analysis suggests that it has its own promoter (Figure 1). To test if forced expression of *sfa3* resulted in increased production of Hcp3, as a readout for the H3-T6SS, miniCTX*sfa3* was integrated into PA14 and PA14*rsmA*. Western blot analysis showed that Hcp3 expression was unaltered upon production of either Sfa3 or Sfa3^myc^ in these strains (Figure 7D). In conclusion we found no overlap in the control associated with Sfa2 and Sfa3 and whereas Sfa2 could be shown to coordinate RpoN-dependent expression of the H2-T6SS genes the role of Sfa3 remains elusive. Additionally, this highlights that a Sfa protein can guide RpoN to drive expression of specific genes and that Sfa2 provides specificity to enable control of the H2-T6SS genes.

## DISCUSSION

How bacteria sense and control their competitors in specific environment is increasingly being shown to be finely balanced for optimal deployment and bacterial gain (1). Controlling the production of a large nanomachine such as the antibacterial T6SS is key to prevent unnecessary energy expenditure in environments where its deployment would not be advantageous, e.g. low nutrient media with low densities of organisms (57). On the other hand, environments with high densities of organisms such as in biofilms, or where bacteria sense competitors or lysis of their kin, expression of competition systems such as the T6SS would be most advantageous. In addition, among the three T6SSs available to *P. aeruginosa*, the H1-T6SSs has been shown to be a defensive type and mostly involved in retaliating to attack in a tit-for-tat mechanism (58). A number of danger signals, including membrane perturbations both endogenous and exogenous have been shown to stimulate T6SS activity (58–61).

With such complexity in the various situations in which the T6SS might be needed, the regulation of these systems has to be complex and multilayered. *P. aeruginosa* is a fine example with a huge network of regulators for expression under various conditions, including c-di-GMP, metal ion levels (iron, molybate, copper), quorum sensing LasR, RhlR, PqsR and VqsR, Fur, CueR, Anr, PsrA, MvaT, MvaU, AmrZ, RetS and RsmA, all somehow intersecting to control the T6SS landscape and indeed some of these global regulators act synergistically and antagonistically upon the T6SS genes (2,3,62–71). These regulators can also act both directly and indirectly to control the expression of these genes. We have previously shown that the post-transcriptional regulator RsmA controls all three T6SS gene clusters in *P. aeruginosa* (3). Here, we build upon this to show a truly global impact upon genes from these three clusters and from all the *vgrG*/*hcp* orphan operons. Combining our observations with direct RsmA binding data from published studies highlights the dual nature of direct and indirect regulation via RsmA (Figure 1) (34,35). Additionally, RsmA/CsrA homologues may have roles in T6SSs in other Gram-negative organisms. Recent work showing that the fourth T6SS in *Yersina pseudotuberculosis* is modulated by this post-transcriptional regulator suggests this mechanism of T6SS control might be widespread to enable bacteria to rapidly respond to changes in their environment (72). Recent studies showing that RsmA binds nascent transcripts as soon as they emerge from the RNA polymerase further blurs the classical distinction between transcriptional and post-transcriptional regulators (35).

As stated, tightly controlling transcription of the T6SS genes is key to limit energy expenditure. Here, we delineate the role of RpoN or sigma 54, a major alternative sigma factor, in controlling the transcription of T6SS genes in *P. aeruginosa*. RpoN has long been linked with controlling nitrogen metabolism, the assimilation of different caron sources and bacterial motility particularly in Pseudomonads but is increasingly being demonstrated to control many aspects of the bacterial cell surface (6,73,74). We show that RpoN acts to divergently control the three T6SS central clusters; causing a modest repression of the H1-T6SS, a clear repressive role over the H3-T6SS but providing an essential role in expression, production, and function of the H2-T6SS. Furthermore, this sigma factor also serves to coordinate the central H2-T6SS gene cluster with the orphan *vgrG* islands linked with the H2-T6SS which is critical for the deployment of the full complement of T6SS tips and their associated effector proteins.

The specific activation of the H2-T6SS by RpoN may indicate that this system has been dedicated to conditions where nitrogen metabolism is high, while other systems may rather respond to other conditions, for example iron limitation for the H3-T6SS (75). Indeed, our data shows that RpoN binding is linked to enhanced expression of the H2-T6SS but repression of components of the H3-T6SS. Whilst less common, there is clear precedent for RpoN binding acting in a repressive manner with four defined classes: overlapping promoter elements (class I), downstream of the promoter in proximity to the start codon (class II), intragenic (class III) and downstream antisense (class IV) that is thought to interfere with convergently transcribing RNA polymerases (48). In our case a clear RpoN peak is located downstream of *clpV3* and upstream of *vgrG3* suggesting that this is repressed through a class II mechanism. The H2-T6SS also exhibits the broadest range of targets which may give *P. aeruginosa* the largest functional range as it delivers: bacterial effectors, eukaryotic effectors, copper acquisition effectors and plays a role in eukaryotic cell internalisation (28,56,63,76,77). In contrast, the H1-T6SS is specialised for anti-bacterial activity (24,78–85) and the H3-T6SS for Iron acquisition (75). Thus, each system might be adapted for specific environmental conditions or prey that triggers one single system and not the others to keep energy consumption to a minimum.

RpoN is also required for expression of flagella and swimming motility. One could speculate that the H2-T6SS may be expressed, over the H1- and H3-T6SS, and assembled in low viscosity environments to coordinate swimming and competition. Indeed, *Proteus mirabilis* has been shown to use its T6SS in the formation of Dienes lines when two actively expanding, motile swarms meet (86). Recent studies in other organisms such as *V. cholerae, Pseudomonas fluorescens* and *Xanthomonas phaseoli* show coordinate regulation or cross talk between the T6SS and flagella systems occurs (11,87,88). RpoN-dependent specificity can also be controlled through the action of accessory proteins such as SFA.

SFA or EBP family members typically form hexamers and are key to activate RpoN/polymerase complexes. Here, we show that Sfa2 provide specificity to this system as it solely controls H2-T6SS genes and serves to refine the focus of RpoN. We show that Sfa2 is critical for gene expression, protein production and functionality of the H2-T6SS. Further the Sfa3 protein, encoded within the H3-T6SS cluster cannot influence the H2-T6SS genes. This corroborates the marked difference in the architecture of Sfa2 and Sfa3. Sfa2 is noticeably longer and contains a GAF domain. Such sensing domains could reflect the environmental conditions which trigger specific induction of the H2-T6SS in a RpoN-dependent manner. However, recent work has shown the SFA homologue encoded within the large T6SS cluster from *V. cholerae*, VasH, acts to detect the intracellular levels of Hcp to control T6SS expression and limit wasteful energy expenditure (89). As the signal for Sfa2 is unknown a similar mechanism could be occurring in *P. aeruginosa*. Indeed, each of our four *vgrG* gene islands with the strongest observed RpoN binding is upstream of a *hcp2* gene (PA14 has 4, Hcp2ABC) and these promoter regions have clear homology suggesting that they are the result of duplications and subsequent diversification. RpoN/Sfa2 control of these clusters would also ensure appropriate levels of Hcp2 are produced at the same time as the central cluster. However, the fact the Sfa3 lacks a GAF domain, or that H1-T6SS is not associated with an SFA, highlights that alternative regulatory mechanisms may exist to control Hcp levels.

RpoN and EBP/SFA control of T6SS clusters may be a widespread mechanism to control the T6SS genes as other *Pseudomonas* species may use SFA proteins to control their T6SS clusters. For example, PSPTO_2549 and PSPTO_5424, found within the HSI-I and HSI-II T6SS clusters of *P. syringae* pv. tomato, encode two potential σ^54^ transcriptional regulators with 57% and 71% identity to Sfa2 (9,90). Indeed, other T6SS-positive Gram-negative bacteria contain promoters with potential RpoN binding sites and putative EBPs/SFAs encoded within the T6SS clusters (10).

We show that Sfa2 is necessary for the expression of T6SS orphan gene clusters. Thus, the action of Sfa2 links the expression of the core cluster with the *vgrG* islands. This appears to be a conserved mechanism with VasH, also shown to be necessary for expression of the two auxiliary clusters (equivalent to the so named orphan clusters in *P. aeruginosa*) in *V. cholerae*. However, and in contrast to what we observed in *P. aeruginosa* VasH is not key for the expression of the central cluster (54). Our RNA-seq data suggests that RpoN and Sfa2 stringently control expression of the *vgrG/hcp* clusters to a higher degree than the core components of the central cluster. This regulatory link provides further evidence that the products of these orphan *vgrG* clusters use the H2-T6SS for their transport as proposed previously (25). As a trimer of VgrG proteins are essential for tip complex formation and subsequent assembly of the T6SS machine, if no VgrGs were produced, this would prevent assembly and firing of the T6SS. Thus, stringent regulation of *vgrG* expression is a fine mechanism of controlling assembly and function of the T6SS. In line with this idea, the ‘onboard checking mechanism’ has recently been proposed whereby only effector-loaded T6SS fires to prevent pointless secretion (91). As each of these *vgrG* islands encoded both a VgrG and an effector protein this would help to ensure loaded T6SS apparatus for volleys of firing.

Encoding a controlling regulator such as SFA within the cluster makes sense from an evolutionary perspective as it could be acquired together with the T6SS genes through horizontal gene transfer. Since RpoN homologues are common in Gram-negative bacteria, once an organism acquires T6SS/*sfa* clusters they could rapidly get integrated for coordinated expression within the new organism.

It would be impossible to highlight all controlling elements that have been proposed for the T6SS. New type of regulators are continuously discovered such as the novel types of hexametric transcription factors such as RovC that controls T6SS in *Y. pseudotuberculosis*, and intersect with the CsrA nutrient-responsive regulator (72). Furthermore, it is clear that the T6SS control is exerted at all levels, including transcriptional, post-transcriptional and post-translational. This provides a range of mechanisms to adapt the systems to specific conditions by modulating the levels of gene expression, protein production, assembly and even firing of the high energy T6SS harpoon until necessary to prevent preemptive firing. Further studies into the regulatory landscape which control essential systems for bacterial survival, defense, aggression, and virulence factors will enable us to understand higher-level control of these networks that make bacteria successful in thriving in such a multitude of environmental and host contexts.

In summary, we dissected the *P. aeruginosa* RsmA/RpoN/SFA network and how it impacts all T6SS players within this organism. We confirm that RsmA has a key role in repressing all three T6SS gene clusters and multiple products expressed from the *vgrG* islands. We demonstrate that RpoN is required for expression of H2-T6SS genes but represses H3- and H1-T6SS. Both RpoN and Sfa2 are required for specific expression of the H2-T6SS cluster and critically the orphan gene islands associated with the H2-T6SS system. Thus, the action of Sfa2 provides specificity and guides the sigma factor RpoN to coordinate expression of the orphan *vgrG* islands with that of the core H2-T6SS cluster. The combined action of these regulators results in the production and assembly of the H2-T6SS machinery, and its full arsenal of effector loaded tip complexes for bacterial gain.

## DATA AVAILABITILTY

Further information, data, requests for resources and/or reagents should be directed to the corresponding authors. The data have been deposited in NCBI’s Sequence Read Archive data base GSE185398.

## Supplementary Material

gkab1254_Supplemental_Files

## Notes

Present address: Thomas E. Wood, Center for Bacterial Pathogenesis, Division of Infectious Diseases, Department of Medicine, Massachusetts General Hospital, Boston, MA, USA; Department of Microbiology, Blavatnik Institute, Harvard Medical School, Boston, MA, USA.
