# Supplementary material for: RpoN/Sfa2-dependent activation of the Pseudomonas aeruginosa H2-T6SS and its cognate arsenal of antibacterial toxins
Source: Nucleic Acids Res. 2021 Dec 20;50(1):227–43. doi: 10.1093/nar/gkab1254 (PMC8855297; doi:10.1093/nar/gkab1254)
Supplement: gkab1254_Supplemental_Files [file gkab1254_Supplemental_Files.zip › Allsopp et al Sup Fig 1-11.pdf]

SUPPLEMENTARY FIGURES 1-11 for

“RpoN/Sfa2-dependent activation of the *Pseudomonas aeruginosa* H2-T6SS and its cognate arsenal of antibacterial toxins”

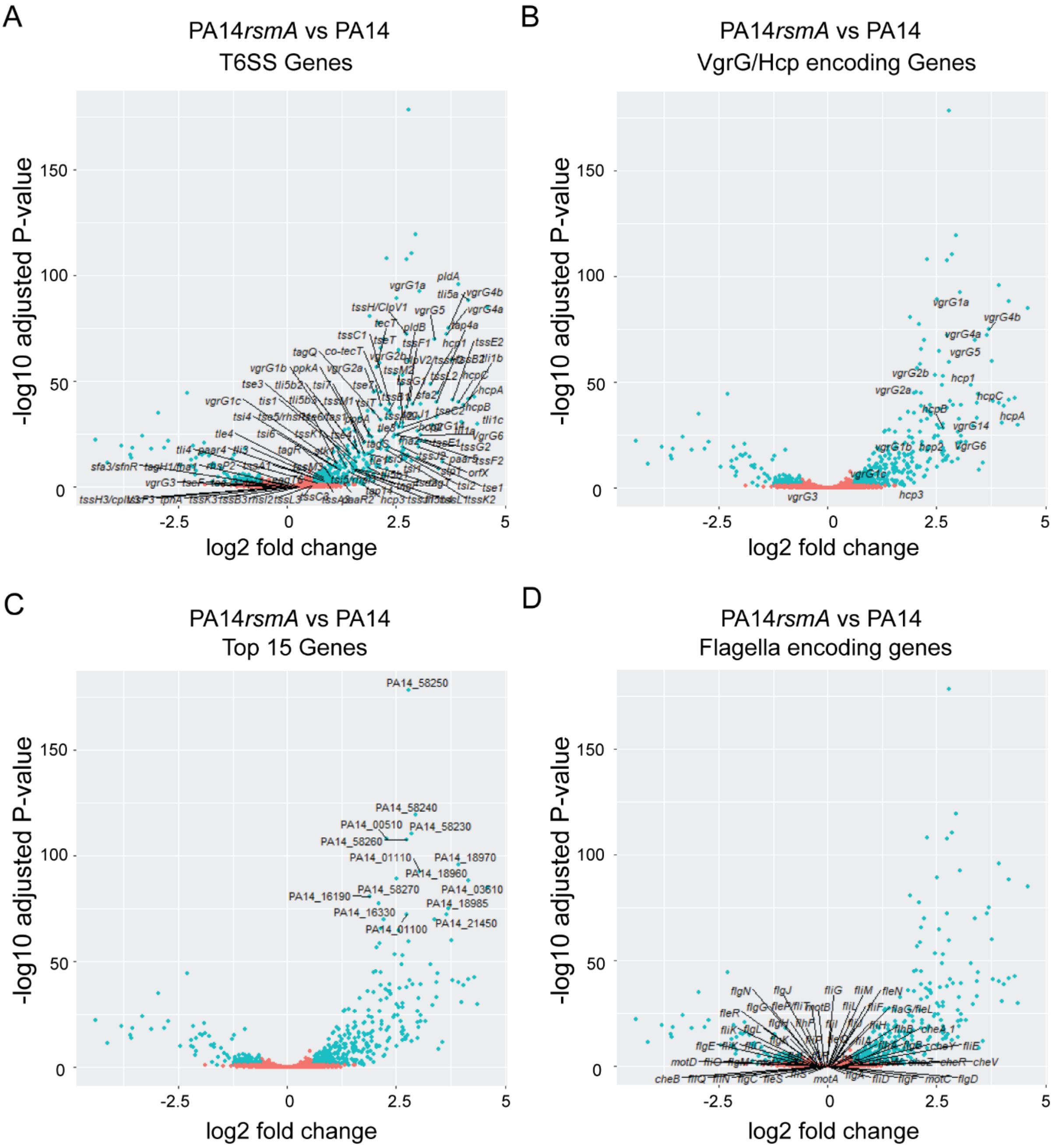

Figure S1: RNA-seq analysis shows extensive RsmA control of T6SS genes. Deletion of *rsmA* results in: (A) activation of T6SS genes from all three T6SSs in *P. aeruginosa*, (B) activation of T6SS VgrG and Hcp genes from all three T6SSs in *P. aeruginosa*. (C) highlighting top 15 most significant hits, (D) no significant effect on genes in the flagella regulon. Volcano plots of differentially expressed genes with colours indicating each gene's absolute log2 (fold change): orange ≤0.58; and blue >0.58 (1.5 fold) with a *P*-adjusted < 0.05 (*n* = 3). Significance was determined by a Wald test and adjusted for multiple comparisons using the Benjamini–Hochberg false-discovery rate correction using DESeq2.

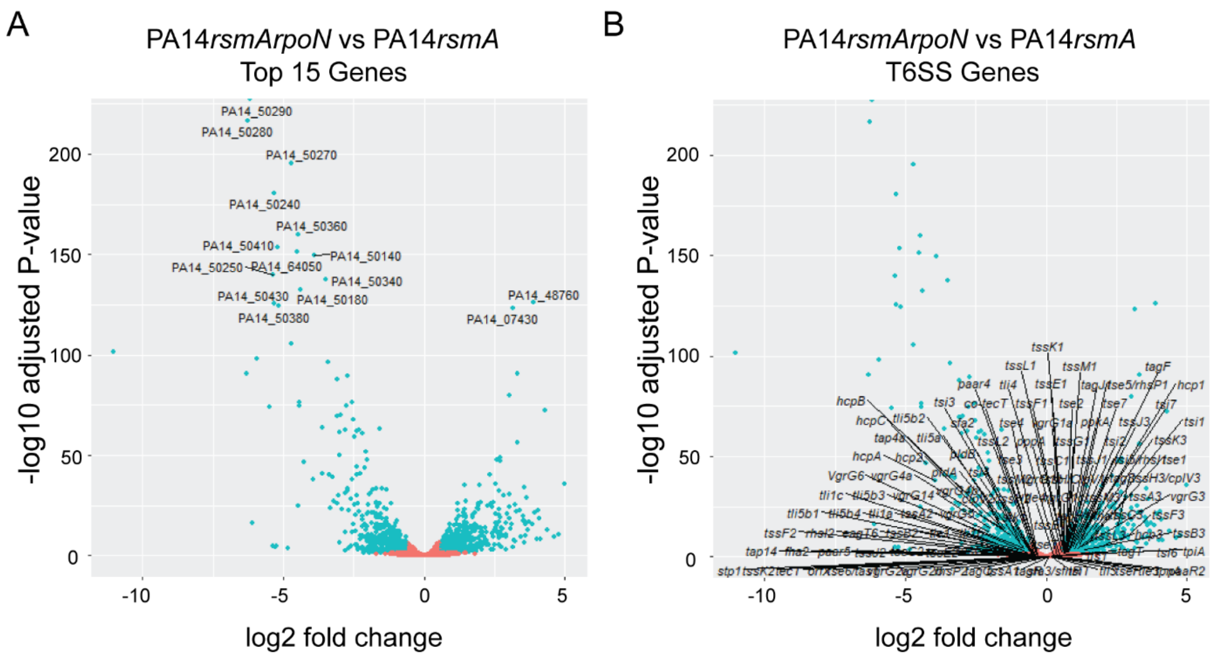

Figure S2: Deletion of RpoN results in altered gene profiles with decreased expression of Flagella genes and H2-T6SS components (A) top 15 most significant hits including 12/15 genes in the Flagella regulon, (B) All T6SS genes labeled highlighting reduced expression of the H2-T6SS genes but enhances expression of multiple genes from the H1- and H3-T6SS. Volcano plots of differentially expressed genes with colours indicating each gene's absolute log2 (fold change): orange ≤0.58; and blue >0.58 (1.5 fold) with a *P*-adjusted <0.05 (n = 3). Significance was determined by a Wald test and adjusted for multiple comparisons using the Benjamini–Hochberg false-discovery rate correction using DESeq2.

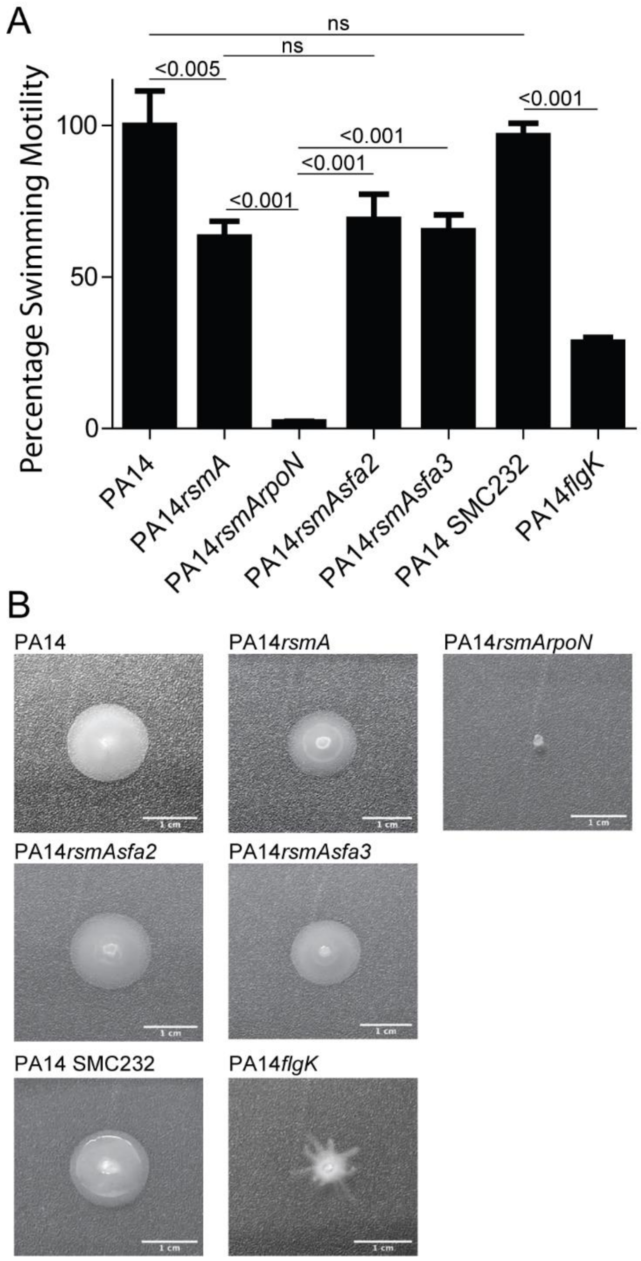

Figure S3: Swimming motility is lost in an *rsmArpoN* mutant but not in an *rsmA*, *rsmAsfa2* or *rsmAsfa3* mutant. (A) Quantification of motility distance expressed as a percentage of PA14. One-way ANOVA with Tukey's multiple comparison test (*n* = 9). (B) Representative images of motility zones for each strain. Published control strains were included in motility assays including PA14 SMC232 and PA14*flgK* (*flgK* encoding the flagella hook protein) (1).

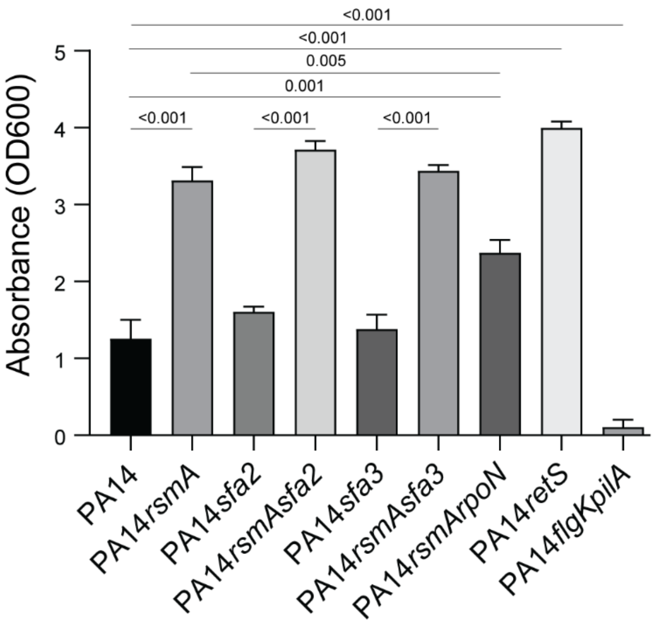

Figure S4: Biofilm formation is reduced in a *rsmArpoN* background but not in a *rsmAsfa2* or *rsmAsfa3* background. Quantification of crystal violet biofilm formation assay. Deletion of *rsmA* enhances biofilm formation as expected. PA14*retS* and PA14*flgKpilA* are included as positive and negative controls. ANOVA with Tukey's multiple comparison test (*n* = 3 biological repeats).

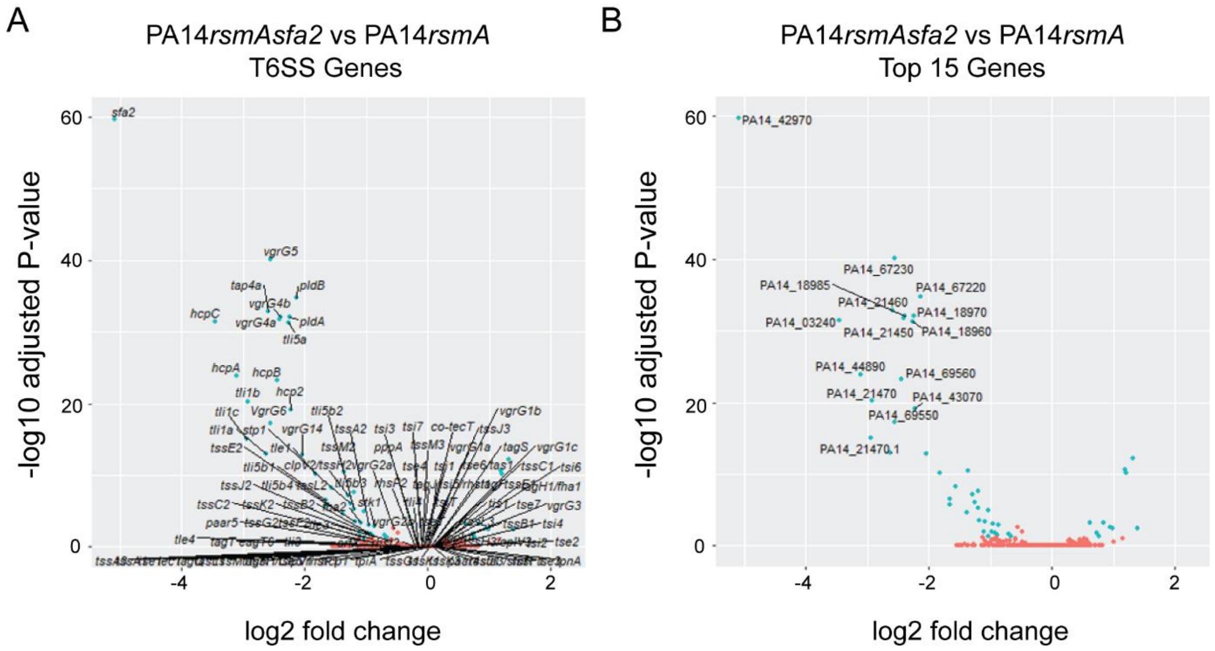

Figure S5: Deletion of *sfa2* results in altered gene profiles with decreased expression of H2-T6SS components. (A) reduced expression of H2-T6SS genes specifically but not those of the H1- of H3-T6SS. (B) top 15 most significant hits all genes associated with the H2-T6SS. Volcano plots of differentially expressed genes with colours indicating each gene's absolute log2 (fold change): orange ≤0.58; and blue >0.58 (1.5 fold) with a *P*-adjusted <0.05 (n = 3). Significance was determined by a Wald test and adjusted for multiple comparisons using the Benjamini–Hochberg false-discovery rate correction using DESeq2.

A

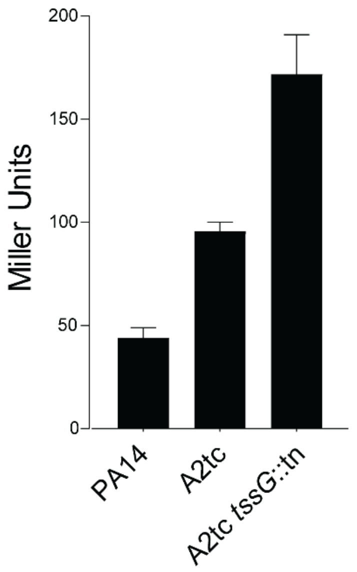

B

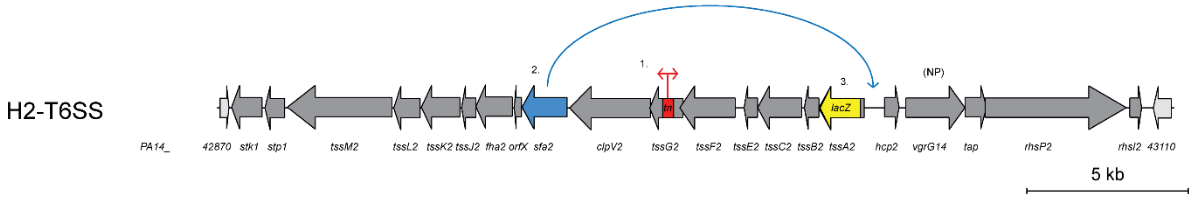

Figure S6: Sfa2 activates the main structural operon via the *tssA2* promoter. (A) Insertion of a transposon with outward facing promoters into *tssG2* results in increased expression of the H2-T6SS *tssA2* transcriptional fusion (A2tc). Graph represents mean +SD (*n* = 3, ANOVA, Tukey's Multiple Comparison Test, *P* <0.005). (B) *tssG2* is located upstream of *sfa2.* Transposon insertion here (1.) would lead to enhanced expression of Sfa2 (2.) resulting in the observed increased activity from the *tssA2* promoter (3.).

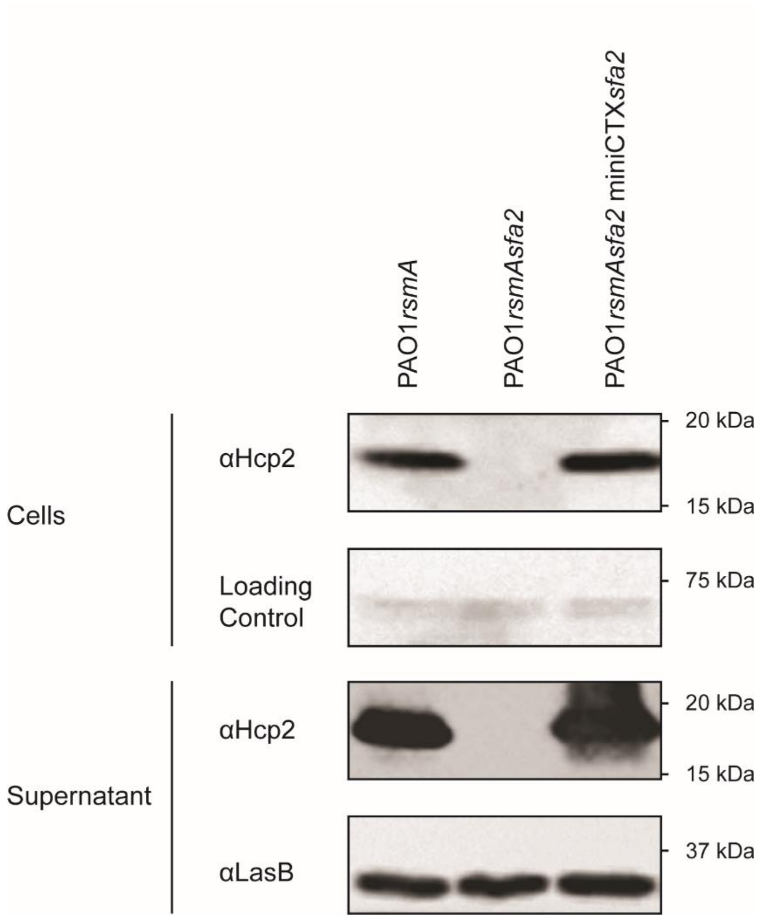

Figure S7: Sfa2 is required for expression and activity of the H2-T6SS in *P. aeruginosa* strain PAO1. Western blot analysis of Hcp2 secretion in a PAO1*rsmA* background using anti-Hcp2 polyclonal sera. A non-specific band detected in the whole cell lysates is used as a cellular loading control, while anti-elastase (LasB) is used as a supernatant loading control.

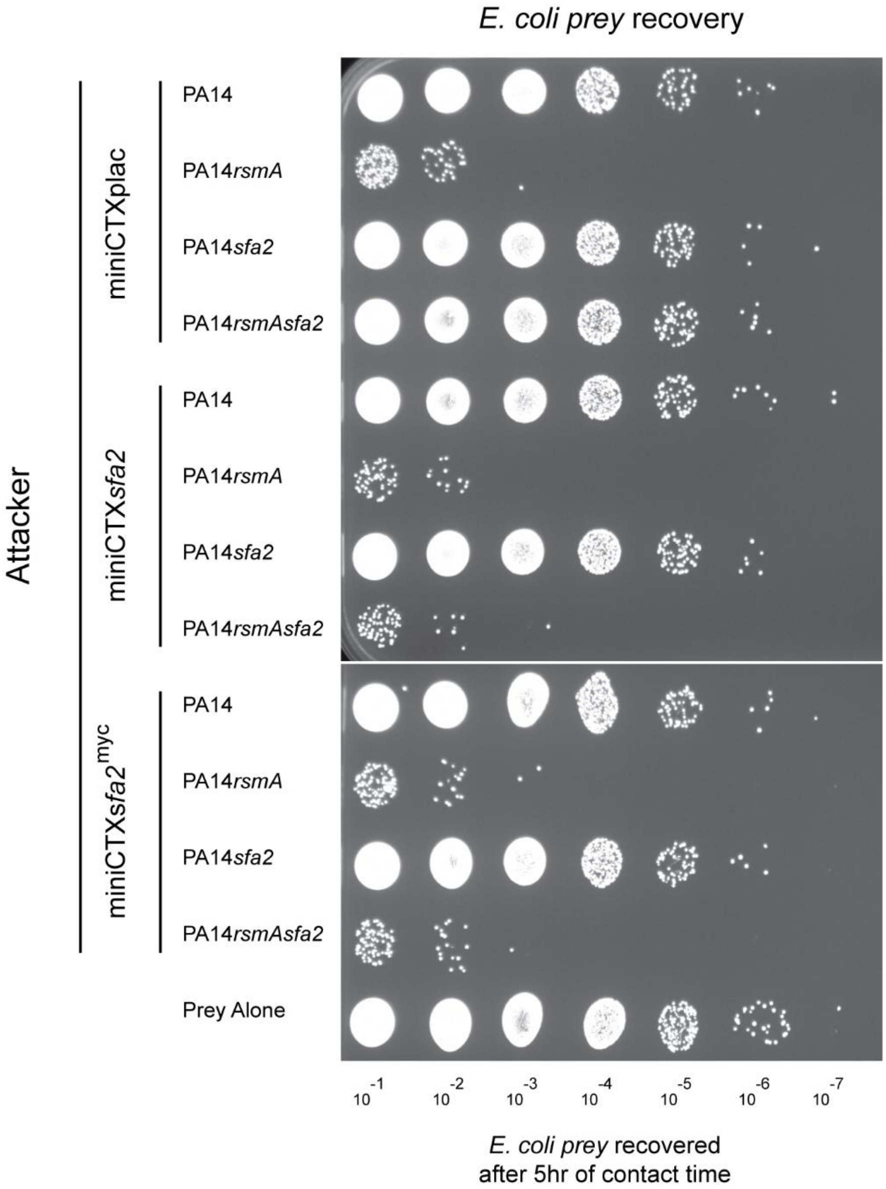

Figure S8: Sfa2 is required for H2-T6SS dependent killing of *E. coli.* Sfa2 is required for H2-T6SS killing as deletion of *sfa2* in a PA14*rsmA* background completely abrogates killing. Complementation of *sfa2* or $sfa2^{myc}$ fully restores killing. Overproduction of *sfa2* in parallel to the WT copy of *sfa2* does not result in enhanced killing. Killing assays was performed for five hours after which the *E. coli* Top10 pGFP+ prey was selected on gentamycin plates. Quantification of killing in Figure 5B.

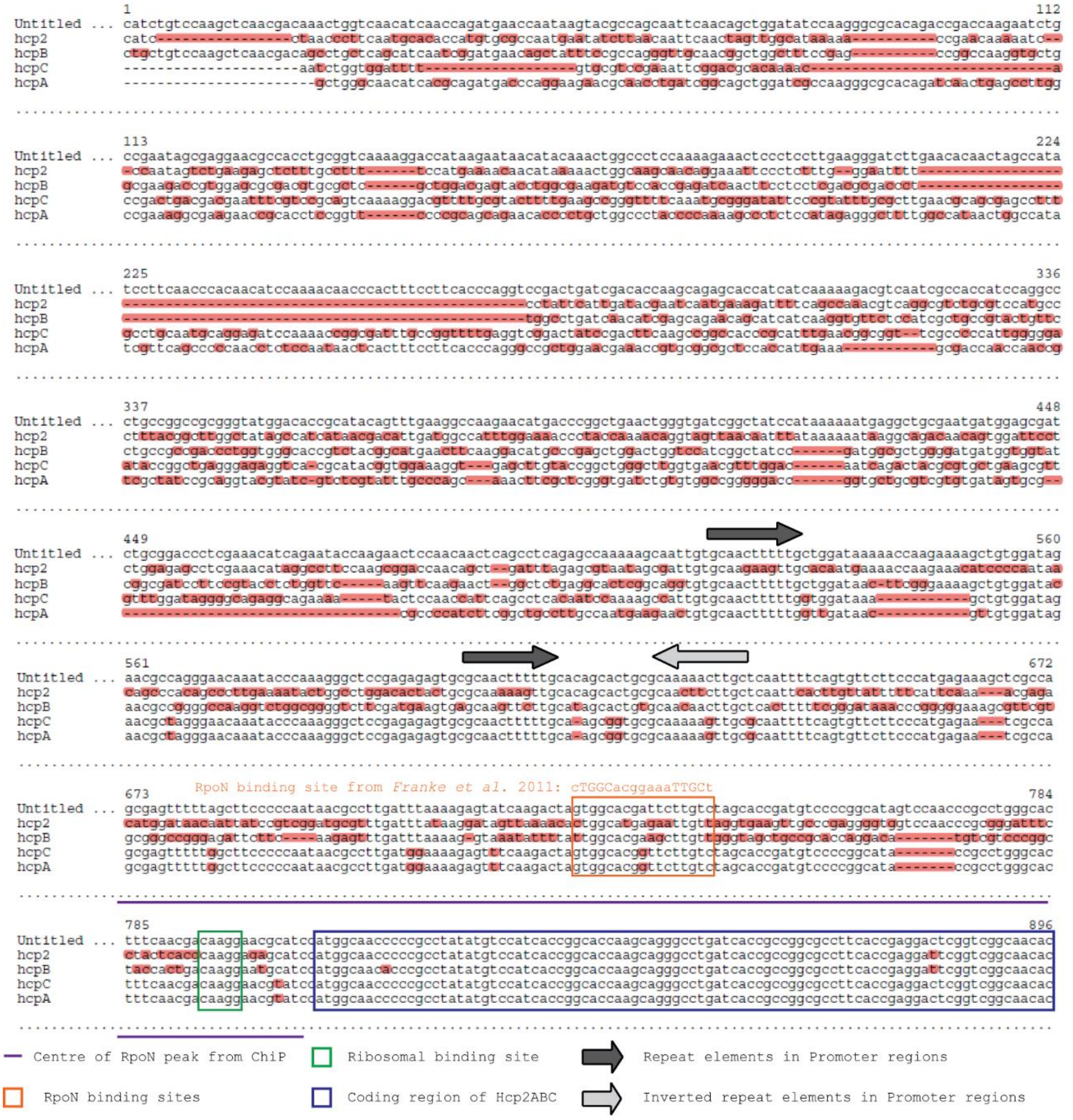

Figure S9: Alignment of H2-T6SS promoter regions with strong RpoN binding reveals high degree of homology, repeat elements and clear RpoN binding motifs. A) Alignment of the promoter regions of *hcp2*, *hcpA*, *hcpB* and *hcpC* shows conservation in sequences with conserved Ribosomal binding sites and start codons. The regions with the highest RpoN enrichment from the ChIP-seq are indicated. Clear RpoN-binding consensus sequences are indicated and are well conserved between the four sequences. Repeat and inverted repeat elements are shown which may be sites of additional regulatory control and may be additional Sfa2 binding sites such as those used by VasH (2).

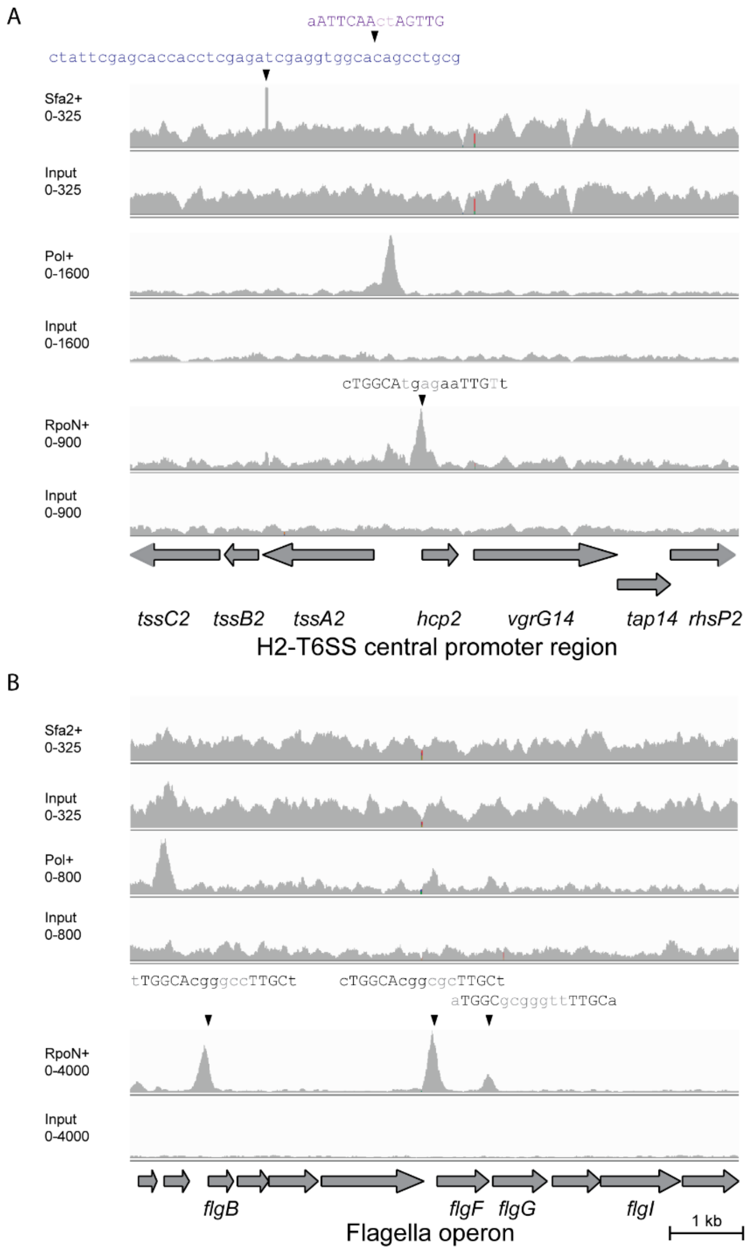

Figure S10: The EBP Sfa2 works in conjunction with RpoN to recruit RNA polymerase and activate expression of the H2-T6SS. ChIP-seq binding profiles identifies a Sfa2 binding in proximity to a RpoN binding and Polymerase binding within the H2-T6SS operon. A potential IHF-binding site (purple) matching the published consensus sequence a(A/t)(A/t)(T/a)CAAxaAgTT(A/g) (3) was identified between the Sfa2 (blue) and RpoN (black) sites which could facilitate DNA bending to enable interaction. The top track for each protein in the Integrative Genome Viewer image shows the binding profiles (Polymerase, RpoN and Sfa2) in proximity to genes of interest. Capitalisation in binding motif indicates the most highly conserved residues identified previously

(3,4). Dark bases indicate optimal residue compared to previously published binding motif and pale shaded bases indicate divergence. A track with the input DNA (input) is show as a negative control. A region of the flagella operon covering *flgB* to *flgI* with clear polymerase and RpoN but no Sfa2 binding is included as a control.

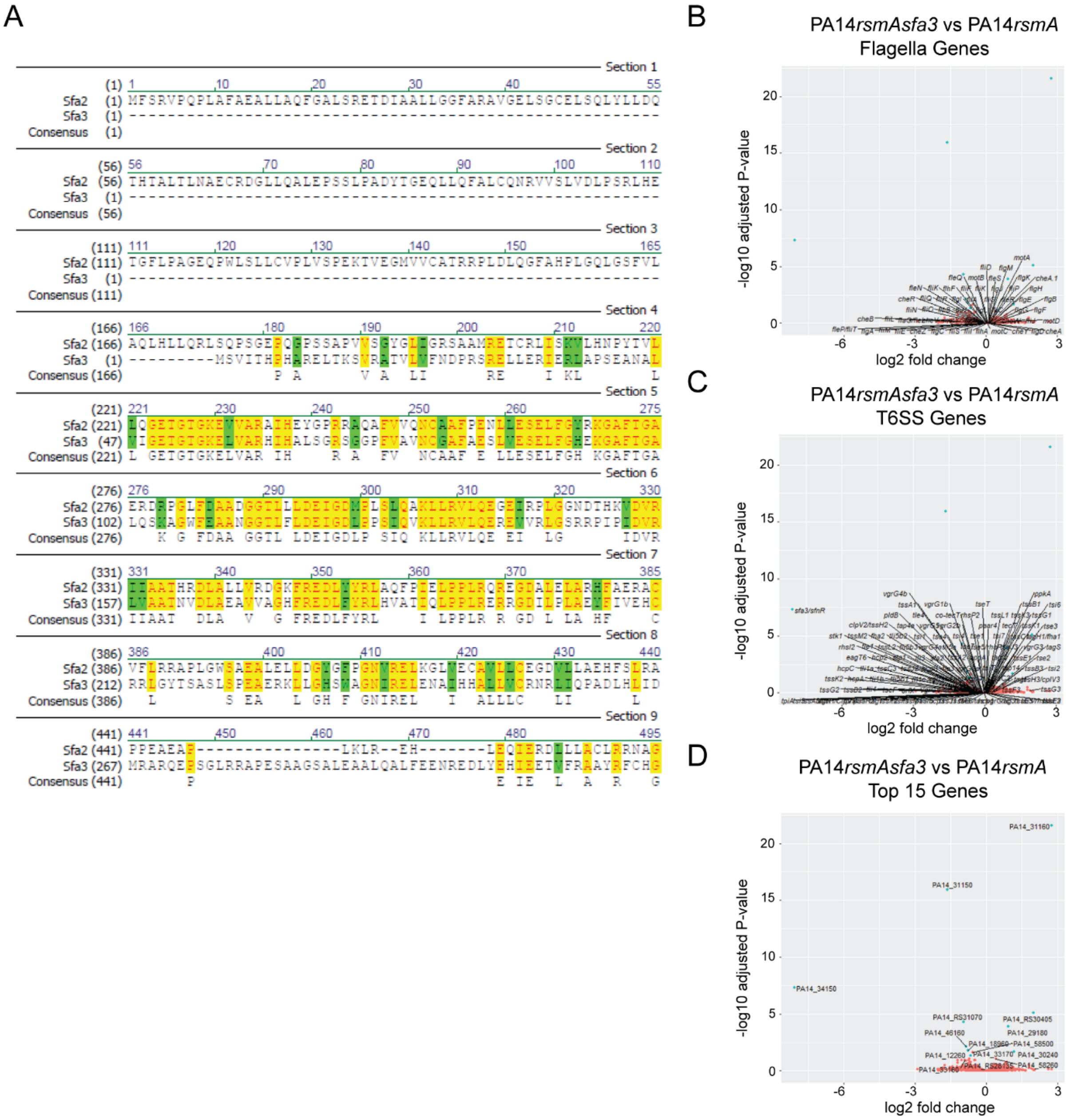

Figure S11: Sfa3 is an EBP but does not mediate control of the T6SSs of *P. aeruginosa* under the conditions tested. (A) Alignment of Sfa2 and Sfa3 showing conserved Sigma 54 interaction domains and absence of N-terminal GAF domain in Sfa3. (B) Deletion of *sfa3* does not result in a significant effect on genes in the Flagella regulon. (C) Deletion of *sfa3* has no clear effect on the T6SS genes in *P. aeruginosa* with the exception of *tli5a* with a modest reduction in expression of -1.69 fold (D) Top 15 most significant hits in a *sfa3* mutant. Volcano plots of differentially expressed genes with colours indicating each gene’s absolute log2 (fold change): orange ≤0.58;

and blue >0.58 (1.5 fold) with a *P*-adjusted < 0.05 (*n* = 3). Significance was determined by a Wald test and adjusted for multiple comparisons using the Benjamini–Hochberg false-discovery rate correction using DESeq2.

Additional References for Supplementary Figures

1. Limoli, D.H., Warren, E.A., Yarrington, K.D., Donegan, N.P., Cheung, A.L. and O'Toole, G.A. (2019) Interspecies interactions induce exploratory motility in Pseudomonas aeruginosa. *eLife*, **8**, e47365.
2. Seibt, H., Aung, K.M., Ishikawa, T., Sjostrom, A., Gullberg, M., Atkinson, G.C., Wai, S.N. and Shingler, V. (2020) Elevated levels of VCA0117 (VasH) in response to external signals activate the type VI secretion system of Vibrio cholerae O1 El Tor A1552. *Environ Microbiol*, **22**, 4409-4423.
3. Goodrich, J.A., Schwartz, M.L. and McClure, W.R. (1990) Searching for and predicting the activity of sites for DNA binding proteins: compilation and analysis of the binding sites for Escherichia coli integration host factor (IHF). *Nucleic Acids Res*, **18**, 4993-5000.
4. Francke, C., Groot Kormelink, T., Hagemeijer, Y., Overmars, L., Sluijter, V., Moezelaar, R. and Siezen, R.J. (2011) Comparative analyses imply that the enigmatic Sigma factor 54 is a central controller of the bacterial exterior. *BMC Genomics*, **12**, 385.
