# Supplementary material for: RpoN/Sfa2-dependent activation of the Pseudomonas aeruginosa H2-T6SS and its cognate arsenal of antibacterial toxins
Source: Nucleic Acids Res. 2021 Dec 20;50(1):227–43. doi: 10.1093/nar/gkab1254 (PMC8855297; doi:10.1093/nar/gkab1254)
Supplement: gkab1254_Supplemental_Files [file gkab1254_Supplemental_Files.zip › Allsopp et al Tables S1 - S3.pdf]

SUPPLEMENTARY TABLES

Table S1. Bacterial strains used in this study.

Name	Description	Source
Escherichia coli		
DH5α	F– *end*A1 *gln*V44 *thi*-1 *rec*A1 *rel*A1 *gyr*A96 *deo*R *nup*G *pur*B20 φ80d*lac*ZΔM15 Δ(*lac*ZYA-*arg*F)U169 *hsd*R17(rK–mK+) λ–	(1)
Top10	F$^-$ *mcrA* Δ(*mrr-hsdRMS-mcrBC*) φ80*lacZ*ΔM15 Δ*lacX74 nupG recA*1 *araD139* Δ(*ara-leu*)7697 *galE15 galK16 rpsL*(Str^R) *endA1* λ$^-$	Invitrogen
CC118λ*pir*	*ara*D Δ(*ara*, *leu*) Δ*lac*Z74 *pho*A20 *gal*K *thi*-1 *rsp*E *rpo*B *arg*E *rec*A1 λ*pir*	(2)
1047	Host strain for pRK2013	(3)
BL21(DE3)	F– *omp*T *gal dcm lon hsd*SB(rB–mB–) λ(DE3 [*lac*I *lac*UV5-T7p07 *ind*1 *sam*7 *nin*5]) [*mal*B+]K-12(λS)	(4)
Pseudomonas aeruginosa		
PA14	Wild-type	(5)
PA14*rsmA*	Markerless mutant	(6)
PA14*rsmArpoN*	Markerless mutant	This study
PA14*sfa2*	Markerless mutant	This study
PA14*rsmAsfa2*	Markerless mutant	This study
PA14*sfa3*	Markerless mutant	This study
PA14*rsmAsfa3*	Markerless mutant	This study
PA14*retS*	Markerless mutant	(6)
PA14 A2tc	PA14*tssA2*::*lacZ*tc (*tssA2 lacZ* transcriptional fusion) Markerless mutant	(6)
PA14 A2tc *rsmA*	PA14*tssA2*:: *lacZ*tc (*tssA2 lacZ* transcriptional fusion) Markerless mutant	(6)
PA14 A2tc *rsmArpoN*	PA14*tssA2*:: *lacZ*tc (*tssA2 lacZ* transcriptional fusion) Markerless mutant	This study
PA14 A2tc *tssG2*::tn	Transposon insertion into *tssG2*, Gm^R	(6)
PA14 B3tc	PA14*tssB3*::*lacZ*tc (*tssB3 lacZ* transcriptional fusion) Markerless mutant	This study
PA14 B3tc *rsmA*	PA14*tssB3*::*lacZ*tc (*tssB3 lacZ* transcriptional fusion) Markerless mutant	This study
PA14 B3tc *rsmArpoN*	PA14*tssB3*::*lacZ*tc (*tssB3 lacZ* transcriptional fusion) Markerless mutant	This study
PA14*rsmA* H2-T6SS	*tssA2-sfa2*, Markerless mutant	This study
PA14 SMC232	O'Toole Laboratory Wild-type	(5,7)
PA14*flgK*	Markerless mutant	(7,8)
PA14*flgKpilA*	Markerless mutant	(7,8)
PAO1	Wild-type	(9)
PAO1*rsmA*	Markerless mutant	(9)
PAO1*rsmAsfa2*	Markerless mutant	This study

Table S2. Plasmids used in this study.

Name	Description	Source
pCR-BluntII-TOPO	Cloning vector, ColE1 *ori*, Kan^R	Invitrogen
pRL662-*gfp*	Broad host range vector derived from pBBR1MCS-5 expressing GFP2, Gm^R	Erh-Min Lai collection Used (6)
pRK2013	Helper plasmid, ColE1 *ori*, *mob*RK2, *tra*RK2, Kan^R	(10)
pKNG101	Gene replacement suicide vector, R6K *ori*, *sacB*, Str^R	(11)
pKNG101-*rsmA*	Gene deletion construct for *rsmA* (PA14_52570)	(6)
pKNG101-*rpoN*	Gene deletion construct for *rpoN* (PA14_57940)	This study
pKNG101-H2	Gene deletion construct for *tssA2*-*sfa2* (PA14_43050-PA14_42970)	(12)
pKNG101-*sfa2*	Gene deletion construct for *sfa2* PA14_42970	This study
pKNG101-*sfa3*	Gene deletion construct for *sfa3* PA14_34150	This study
pKNG101-*retS*	Gene deletion construct for *retS* PA14_64230	(6)
pKNG101-$rpoN^{Flag}$	*rpoN* with C-terminal triple Flag tag at native locus (PA14_57940)	This study
pKNG101-$sfa2^{Flag}$	*sfa2* with C-terminal triple Flag tag at native locus (PA14_42970)	This study
miniCTXplac	Plasmid for the integration of genes into the *att* site of pseudomonal chromosomes, pMB1-derived *ori*, P*lac*, Tet^R	(13)
miniCTX*rpoN*	*rpoN* cloned into miniCTXplac, Tet^R	This study
miniCTX*sfa2*	*sfa2* cloned into miniCTXplac, Tet^R	This study
miniCTX$sfa2^{myc}$	*sfa2* cloned into miniCTXplac, C-terminal fusion to myc tag, Tet^R	This study
miniCTX*sfa3*	*sfa3* cloned into miniCTXplac, Tet^R	This study
miniCTX$sfa3^{myc}$	*sfa3* cloned into miniCTXplac, C-terminal fusion to myc tag, Tet^R	This study

Table S3

Number	Brief Description	Sequence	Source
OAL2280	rsmA_Ext_F	GTCGAGTTCGCCGGCAAGTACAAC	(6)
OAL2281	rsmA_Ext_R	CTTTCGGTATGGCGCACTCAGG	(6)
OAL4405	rpoN_Up_F BamHI	AGTCAAGGATCCATTCATCCTCCTCGACGAGCCCTTC	This study
OAL4406	rpoN_Up_R	ACCAGTCGCGATGGTTTCATGGCTG	This study
OAL4407	rpoN_Down_F	AAACCATCGCGACTGGTGTGACGTT	This study
OAL4408	rpoN_Down_R ApaI	TTGACTGGGCCCCGATTTGTTCGAGTACGCGTTTCTT	This study
OAL4409	rpoN_Ext_F	CTACCTGCCGCAGGAAGCCTCGATC	This study
OAL4410	rpoN_Ext_R	AAGTCCACGGGAGCATCCAGGTGC	This study
OAL3843	tssA2_Ext_F (H2- deletion)	GCTCTCCTCGCATTATCTGGAACTCGC	This study
OAL3844	sfa2_Ext_R (H2-deletion)	CAGCTCGCTTTCCAGCAGGTTTTCC	This study
OAL3151	sfa2_Up_F	ATACCTTGATCCTGATGACCTCCAA	This study
OAL3152	sfa2_Up_R	TCGTCGCGGGCTGAACATCGCACCCAC	This study
OAL3153	sfa2_Down_F	ATGTTCAGCCCGCGACGAACCCTGATCTA	This study
OAL3154	sfa2_Down_R	CTGGTGTCGGTCAGGTAGAAC	This study
OAL3155	sfa2_Ext_F	GGACGTGCTCAACCTGTTCT	This study
OAL3156	sfa2_Ext_R	ACTTTCCACGGGTTCCTGC	This study
OAL4857	sfa3_Up_F	TAGTAGGATCCCGCCTGGAACAGTGGTGCG	This study
OAL4858	sfa3_Up_R	CAGCGAGACGGGATGGGTGATGACGGACAT	This study
OAL4859	sfa3_Down_F	ACCCATCCCGTCTCGCTGGTGGGCAAGTGA	This study
OAL4860	sfa3_Ext_F	GCTGCTGGCGGAACTCTGC	This study
OAL1897	retS_Ext_F	GAGGAGGCCAGCTTCATCGTCATG	(6)
OAL1898	retS_Ext_R	TGCTGCACGTTGTCGCTCTG	(6)
OAL4852	rpoNFlag_Up_F BamHI	TAGTAGGATCCGCTGGTCGAGCTGAACCAGG	This study
OAL4853	rpoNFlag _Up_R	GTCGTCATCTTTGTAGTCGATATCATGATCTTTATAATCACCGTCATGG TCTTTGTAGTCCACCAGTCGCTTGCGCTCG	This study
OAL4854	rpoNFlag _Down_F	ATCGACTACAAAGATGACGACGATAAATGACGTTGATCCACGCCAAG GT	This study
OAL4855	rpoNFlag _Down_R SmaI	TAGTACCCGGGCGTCGAACTCCGGCAGCTC	This study
OAL4856	rpoNFlag _Ext_F	CCACGCCCTGGCTGAACG	This study
OAL4841	sfa2Flag_Up_F BamHI	TAGTAGGATCCGTGTTGCAGGAAGGCGAGATTCG	This study
OAL4842	sfa2 Flag _Up_R	GTCGTCATCTTTGTAGTCGATATCATGATCTTTATAATCACCGTCATGG TCTTTGTAGTCGGTCCGGGGATCGCCGAAAT	This study
OAL4843	sfa2 Flag _Down_F	ATCGACTACAAAGATGACGACGATAAATGAAGCCCATGATCGAGATTT CCTTCCA	This study
OAL4844	sfa2 Flag _Down_R SmaI	TAGTACCCGGGGCATCGTCCGGAATGATGCTGC	This study
OAL4845	sfa2 Flag _Ext_F	TACCGGCAAGGAGGTGGTCG	This study
OAL4846	sfa3Flag_Up_F BamHI	TAGTAGGATCCCTTCCGCGAGGACCTGTTCTACC	This study
OAL4847	sfa3 Flag _Up_R	GTCGTCATCTTTGTAGTCGATATCATGATCTTTATAATCACCGTCATGG TCTTTGTAGTCCTTGCCCACCAGCGAGACC	This study
OAL4848	sfa3Flag_Down_F	ATCGACTACAAAGATGACGACGATAAATGAGCGCGGCGCTTTACAG	This study
OAL4849	sfa3Flag_Down_R SmaI	TAGTACCCGGGCAACAGTTCAGTTCAG	This study
OAL4850	sfa3Flag_Ext_F	CCTTTACCGGTGCGTTGCAGA	This study
OAL4851	sfa3Flag_Ext_R	GATCAGGGCCTGGGTGAGTACC	This study
OAL4437	rpoNCTX_F BamHI	TAGATGGATCCTAACAGGAGGAATTAACCATGAAACCATCGCTAGTCC TCAAGATG	This study
OAL4438	rpoNCTX_R SacI	TAGATGAGCTCTCACACCAGTCGCTTGCGCTCGC	This study
OAL4440	spoNFseq	GAAGTGGAAGTCGTGCTGCG	This study
OAL3868	sfa2CTX_F BamHI	TAGATGGATCCTAACAGGAGGAATTAACCATGTTCAGCCGCGTACCG CAACC	This study
OAL3869	sfaCTX_R SacI	TAGATGAGCTCTCAGGTCCGGGGATCGCCGA	This study
OAL3871	sfa2CTXmyc_R SacI	TAGATGAGCTCTCACAGGTCCTCCTCGGAGATCAGCTTCTGCTCCAT GGTCCGGGGATCGCCGAAAT	This study

OAL4441	sfa3CTX_F BamHI	TAGATGGATCCTAACAGGAGGAATTAACCATGTCCGTCATCACCCATCCCCAC	This study
OAL4442	sfa3CTX_R SacI	TAGATGAGCTCTCACTTGCCCACCAGCGAGACCA	This study
OAL4443	sfa3CTXmyc_R	TAGATGAGCTCTCACAGGTCCTCCTCGGAGATCAGCTTCTGCTCCATCTTGCCCACCAGCGAGACCA	This study
OAL4444	sfa3Fseq	AGGTCAAGCTGCTGCGCGTG	This study

ADDITIONAL REFERENCES

1. Hanahan, D. (1985) *DNA cloning: a Practical Approach*. IRL Press, McLean, Virginia.
2. Herrero, M., de Lorenzo, V. and Timmis, K.N. (1990) Transposon vectors containing non-antibiotic resistance selection markers for cloning and stable chromosomal insertion of foreign genes in gram-negative bacteria. *Journal of bacteriology*, **172**, 6557-6567.
3. Hachani, A., Allsopp, L.P., Oduko, Y. and Filloux, A. (2014) The VgrG proteins are "a la carte" delivery systems for bacterial type VI effectors. *J Biol Chem*, **289**, 17872-17884.
4. Studier, F.W. and Moffatt, B.A. (1986) Use of bacteriophage T7 RNA polymerase to direct selective high-level expression of cloned genes. *J Mol Biol*, **189**, 113-130.
5. Rahme, L.G., Stevens, E.J., Wolfort, S.F., Shao, J., Tompkins, R.G. and Ausubel, F.M. (1995) Common virulence factors for bacterial pathogenicity in plants and animals. *Science (New York, N.Y.)*, **268**, 1899-1902.
6. Allsopp, L.P., Wood, T.E., Howard, S.A., Maggiorelli, F., Nolan, L.M., Wettstadt, S. and Filloux, A. (2017) RsmA and AmrZ orchestrate the assembly of all three type VI secretion systems in Pseudomonas aeruginosa. *Proc Natl Acad Sci U S A*, **114**, 7707-7712.
7. Limoli, D.H., Warren, E.A., Yarrington, K.D., Donegan, N.P., Cheung, A.L. and O'Toole, G.A. (2019) Interspecies interactions induce exploratory motility in Pseudomonas aeruginosa. *eLife*, **8**, e47365.
8. Ribbe, J., Baker, A.E., Euler, S., O'Toole, G.A. and Maier, B. (2017) Role of Cyclic Di-GMP and Exopolysaccharide in Type IV Pilus Dynamics. *J Bacteriol*, **199**.
9. Wood, T.E., Howard, S.A., Forster, A., Nolan, L.M., Manoli, E., Bullen, N.P., Yau, H.C.L., Hachani, A., Hayward, R.D., Whitney, J.C. *et al.* (2019) The Pseudomonas aeruginosa T6SS Delivers a Periplasmic Toxin that Disrupts Bacterial Cell Morphology. *Cell Rep*, **29**, 187-201 e187.
10. Figurski, D.H. and Helinski, D.R. (1979) Replication of an origin-containing derivative of plasmid RK2 dependent on a plasmid function provided in trans. *Proceedings of the National Academy of Sciences of the United States of America*, **76**, 1648-1652.
11. Kaniga, K., Delor, I. and Cornelis, G.R. (1991) A wide-host-range suicide vector for improving reverse genetics in gram-negative bacteria: inactivation of the blaA gene of Yersinia enterocolitica. *Gene*, **109**, 137-141.
12. Jones, C., Hachani, A., Manoli, E. and Filloux, A. (2014) An rhs gene linked to the second type VI secretion cluster is a feature of the Pseudomonas aeruginosa strain PA14. *J Bacteriol*, **196**, 800-810.
13. Lossi, N.S., Manoli, E., Forster, A., Dajani, R., Pape, T., Freemont, P. and Filloux, A. (2013) The HsiB1C1 (TssB-TssC) complex of the Pseudomonas aeruginosa type VI secretion system forms a bacteriophage tail sheathlike structure. *J Biol Chem*, **288**, 7536-7548.
