# Supplementary material for: RpoN/Sfa2-dependent activation of the Pseudomonas aeruginosa H2-T6SS and its cognate arsenal of antibacterial toxins
Source: Nucleic Acids Res. 2021 Dec 20;50(1):227–43. doi: 10.1093/nar/gkab1254 (PMC8855297; doi:10.1093/nar/gkab1254)
Supplement: gkab1254_Supplemental_Files [file gkab1254_Supplemental_Files.zip › NAR-03535-X-2021 Allsopp Sup Tables List.docx]

**SUPPLEMENTARY TABLES LIST**

**Table S1:** Strains used in this study

**Table S2:** Plasmids used in this study

**Table S3:** Primers used in this study

**Table S4:** RNA-seq results of PA14*rsmA* vr PA14

**Table S5:** RNA-seq results of PA14*rsmArpoN* vr PA14*rsmA*

**Table S6:** RNA-seq results of PA14*rsmAsfa2* vr PA14*rsmA*

**Table S7:** Summary of genome-wide binding sites determined by ChIP-seq

**Table S8:** RNA-seq results of PA14*rsmAsfa3* vr PA14*rsmA*
